# A Decade of Innovation in Breast Cancer (2015–2025): A Comprehensive Review of Clinical Trials, Targeted Therapies and Molecular Perspectives

**DOI:** 10.3390/cancers18030361

**Published:** 2026-01-23

**Authors:** Klaudia Dynarowicz, Dorota Bartusik-Aebisher, Sara Czech, Aleksandra Kawczyk-Krupka, David Aebisher

**Affiliations:** 1 Department of Biochemistry and General Chemistry, Faculty of Medicine, University of Rzeszów, 35-310 Rzeszów, Poland; kdynarowicz@ur.edu.pl (K.D.); dbartusikaebisher@ur.edu.pl (D.B.-A.); 2 English Division Science Club, Collegium Medicum, Faculty of Medicine, University of Rzeszów, 35-310 Rzeszów, Poland; sc126240@stud.ur.edu.pl; 3 Department of Internal Diseases, Angiology and Physical Medicine, Center for Laser Diagnostics and Therapy, Faculty of Medical Sciences in Zabrze, Medical University of Silesia, 40-055 Katowice, Poland; 4 Department of Photomedicine and Physical Chemistry, Faculty of Medicine, University of Rzeszów, 35-310 Rzeszów, Poland

**Keywords:** breast cancer, HR+/HER2− disease, HER2-positive breast cancer, HER2-low breast cancer, triple-negative breast cancer, CDK4/6 inhibitors, PI3K/AKT/mTOR pathway inhibitors, SERD therapy, antibody–drug conjugates, photodynamic and phototheranostic therapies

## Abstract

The past decade has witnessed an unprecedented transformation in breast cancer management, driven by parallel advances in targeted therapies, immunomodulation, drug-delivery technologies, and molecular diagnostic tools. This review summarizes the key achievements of 2015–2025, encompassing all major biological subtypes of breast cancer as well as technological innovations with substantial clinical relevance. In hormone receptor-positive (HR+)/HER2− disease, the integration of CDK4/6 inhibitors, modulators of the PI3K/AKT/mTOR pathway, oral Selective Estrogen Receptor Degraders (SERDs), and real-time monitoring of Estrogen Receptor 1 (ESR1) mutations has enabled clinicians to overcome endocrine resistance and dynamically tailor treatment based on evolving molecular alterations detected in circulating biomarkers. In HER2-positive breast cancer, treatment paradigms have been revolutionized by next-generation antibody–drug conjugates, advanced antibody formats, and technologies facilitating drug penetration across the blood–brain barrier, collectively improving systemic and central nervous system disease control. The most rapid progress has occurred in triple-negative breast cancer (TNBC), where synergistic strategies combining selective cytotoxicity via Antibody-Drug Conjugates (ADCs), DNA damage response inhibitors, immunotherapy, epigenetic modulation, and therapies targeting immunometabolic pathways have markedly expanded therapeutic opportunities for this historically challenging subtype. In parallel, photodynamic therapy has emerged as an investigational and predominantly local phototheranostic approach, incorporating nanocarriers, next-generation photosensitizers, and photoimmunotherapy capable of inducing immunogenic cell death and modulating antitumor immune responses. A defining feature of the past decade has been the surge in patent-driven innovation, encompassing multispecific antibodies, optimized ADC architectures, novel linker–payload designs, and advanced nanotechnological and photoactive delivery systems. By integrating data from clinical trials, molecular analyses, and patent landscapes, this review illustrates how multimechanistic, biomarker-guided therapies supported by advanced drug-delivery technologies are redefining contemporary precision oncology in breast cancer. The emerging therapeutic paradigm underscores the convergence of targeted therapy, immunomodulation, synthetic lethality, and localized immune-activating approaches, charting a path toward further personalization of treatment in the years ahead.

## Simple Summary

Over the past decade, breast cancer treatment has advanced more rapidly than ever before. New targeted medicines improved immune-based therapies, modern drug-delivery technologies, and highly precise molecular diagnostic tools have made it possible to tailor treatment to the unique biology of each tumor. In this review, we summarize the major progress made between 2015 and 2025 across all main types of breast cancer, including modern hormone therapies, drugs that block key growth pathways, treatments directed at the Human Epidermal Growth Factor Receptor 2 (HER2) protein, immunotherapies, antibody–drug conjugates, as well as emerging and primarily adjunctive photodynamic approaches. We explain how discoveries from clinical trials, molecular research, and technology-driven innovation are reshaping current and emerging treatment paradigms. Understanding these advances can help guide the development of more effective and personalized therapies, ultimately improving outcomes for people diagnosed with breast cancer.

## 1. Introduction

Breast cancer remains one of the most frequently diagnosed malignancies worldwide and represents a major public health challenge. According to GLOBOCAN 2022 estimates, 2,296,840 new cases were reported, accounting for 24% of all cancer diagnoses. In terms of incidence, breast cancer is surpassed only by lung cancer, for which 2,480,675 cases were recorded during the same period. Breast cancer also continues to be the leading cause of cancer-related mortality among women, with 666,103 deaths reported in 2022—representing 15% of all oncologic deaths. Within the female population, it accounts for the highest incidence (557,532 cases) and mortality (144,439 deaths) among all tumor types [1]. In Poland, breast cancer ranks first in both incidence and mortality among women. Between 2013 and 2022, an average of 19.4 thousand new cases were recorded annually, 99.2% of which occurred in women. Epidemiological trends indicate a steady, though moderate, increase in new diagnoses, with the exception of 2020, when a decline was observed—most likely attributable to reduced access to diagnostic and therapeutic services during the COVID-19 pandemic. Currently, the annual number of newly diagnosed cases in Poland exceeds 21 thousand [2,3]. In men, breast cancer remains rare and does not carry epidemiological significance at the population level. Age continues to be one of the primary risk factors for disease development. Although breast cancer most commonly affects postmenopausal women, recent years have shown a growing number of cases in younger age groups, including those under 40 years of age [4]. Despite substantial advancements in molecular and imaging diagnostics, as well as in available therapeutic modalities, breast cancer remains one of the most significant health challenges in both developed and developing countries.

At the molecular level, the ERBB receptor family plays a pivotal role in the initiation and progression of breast cancer, with its dysregulation serving as a starting point for numerous signaling cascades implicated in carcinogenesis. The ERBB family of receptors (HER1/EGFR, HER2/ERBB2, HER3/ERBB3, HER4/ERBB4) constitutes a central regulatory network governing proliferation, differentiation, and survival of mammary epithelial cells. Activation of these receptors through homo- or heterodimerization triggers intracellular signaling cascades, including the RAS/MAPK and PI3K/AKT/mTOR pathways, both of which are fundamental drivers of tumorigenesis. Individual ERBB members exhibit distinct biological properties—HER1 (EGFR) is an active tyrosine kinase frequently implicated in basal-like carcinogenesis; HER3 possesses limited catalytic activity but strongly engages PI3K; whereas HER4 demonstrates complex, tissue-specific functions [5,6]. Among these receptors, HER2 holds particular clinical relevance. Lacking a known ligand, HER2 becomes constitutively active when ERBB2 gene amplification or protein overexpression occurs, thereby promoting unchecked proliferative signaling. This makes HER2 one of the most critical targets in molecularly directed breast cancer therapy [7,8]. Given the vast body of clinical evidence and the central role of HER2 in both the pathogenesis and treatment of breast cancer, the present review places special emphasis on HER2-driven signaling and its intersections with other oncogenic pathways.

The variability in prognosis and clinical behavior of breast cancer reflects its profound biological heterogeneity. Disease development and dynamics are shaped not only by histopathological features but also by complex molecular determinants. Over the past two decades, the biological classification of breast cancer has undergone a substantial evolution—from traditional systems based solely on histologic assessment to advanced models integrating gene-expression profiling together with steroid hormone receptor and HER2 status [9]. Pioneering molecular studies demonstrated that breast cancer is not a uniform disease entity but comprises several distinct biological subtypes, each characterized by specific patterns of signaling pathway activation and differing proliferative potential [10]. Gene-expression analyses have enabled the identification of four major molecular subtypes: luminal A, luminal B, HER2-enriched, and basal-like [11]. The luminal A subtype is marked by a low proliferative index and favorable prognosis, whereas luminal B exhibits higher proliferative activity and a less favorable clinical outcome compared with luminal A. The HER2-enriched subtype is defined by increased activation of the ERBB2 signaling axis and a more aggressive biological phenotype. In contrast, the basal-like subtype most commonly corresponds to TNBC and is associated with high proliferative dynamics and poor prognosis [12].

In clinical practice, due to the availability of immunohistochemical markers, a simplified classification is commonly used, distinguishing three major groups: HR+/HER2−, HER2-positive, and TNBC, which reflect the fundamental biological and prognostic differences among tumors [13]. In recent years, an additional category—HER2-low—has been delineated, encompassing tumors with low HER2 expression (IHC 1+ or 2+/ISH–). Although these malignancies were originally classified as HER2-negative, accumulating molecular evidence indicates that they represent a distinct biological subtype with a unique expression profile [14,15]. Emerging reports also describe an HER2-ultralow subtype, suggesting that further refinement of breast cancer biological classification may more accurately capture its underlying complexity [16].

Between 2015 and 2025, clinical research in breast cancer underwent a fundamental transformation, shifting from traditional trial designs based on histoclinical characteristics to studies closely aligned with the tumor’s molecular biology. Increasingly, these trials incorporated genetic and transcriptomic biomarkers—such as PIK3CA mutations, ESR1 alterations, and defects in DNA damage–repair genes (BRCA1/2)—which enabled more refined patient stratification and improved prediction of therapeutic outcomes [17,18]. In parallel, advances in “omics” technologies and investigations of circulating tumor DNA (ctDNA) have opened new avenues for monitoring minimal residual disease and facilitating the early detection of recurrence [19].

The aim of this review is to present an overview of the most significant advances in breast cancer treatment between 2015 and 2025, with particular emphasis on the role of clinical trials, the development of targeted therapies, and the evolving molecular landscape. The analysis encompasses both the shifts in the biological classification of the disease and the influence of biomarkers on trial design, providing insight into how the past decade has shaped the current and emerging therapeutic paradigms in breast cancer.

## 2. Materials and Methods

The objective of this work was to collect, compare, and critically analyze clinical trials, scientific publications, and patent filings related to advances in breast cancer therapy over the past decade, with particular emphasis on innovations in the treatment of HR+/HER2−, HER2-positive, and TNBC subtypes, as well as modern forms of photodynamic therapy. The literature and patent search was conducted using standardized criteria and encompassed materials published or registered between 1 January 2015, and 31 December 2025.

Four databases were used to identify relevant sources: PubMed, Google Scholar, ClinicalTrials.gov, and Google Patents. Each database was queried using combinations of medical subject headings (MeSH) and free-text terms. The most frequently applied search phrases included: Breast cancer; HR+/HER2− disease; HER2-positive breast cancer; HER2-low breast cancer; Triple-negative breast cancer; CDK4/6 inhibitors; PI3K/AKT/mTOR pathway inhibitors; SERD therapy; Antibody–drug conjugates; Photodynamic and phototheranostic therapies. Search terms were adjusted individually to the requirements of each platform to ensure optimal sensitivity and specificity.

In PubMed and Google Scholar, the search included publications focused on breast cancer therapies, clinical trials, tumor molecular biology, innovative drug technologies, and translational research. Only articles available in English or accompanied by reliable translations were included. Non-relevant materials—such as publications unrelated to therapeutic strategies, purely technical reports not connected to breast cancer, or studies with unverifiable data—were excluded.

In ClinicalTrials.gov, only phase II and III interventional studies involving systemic breast cancer treatment were analyzed. Trials concerning diagnostics, quality of life, imaging, or supportive care were excluded. Completed, active, suspended, and withdrawn trials were evaluated, taking into account available final results, interim data, or protocol descriptions. Particular attention was given to studies investigating CDK4/6 inhibitors, PI3K/AKT/mTOR–targeted agents, anti-HER2 therapies, immunotherapy, DNA damage response (DDR) inhibitors, ADCs, and photodynamic therapies.

In Google Patents, patent filings relevant to breast cancer treatment technologies were examined, including antibody architectures, ADC platforms (novel linker and payload designs), molecules targeting HER2, TROP2, and other antigens, kinase inhibitors, nano- and bioengineering solutions, drug delivery technologies, and photosensitizers employed in PDT. Patents were assessed for their translational relevance and technological innovation potential, with clinical efficacy evaluated exclusively on the basis of published clinical trial data. Documents of purely industrial character, those without biological relevance, unrelated to breast cancer, or lacking a clear therapeutic connection, were excluded.

The search results underwent two-stage screening: initial evaluation of titles and abstracts, followed by full-text assessment or in-depth review of patent documentation. Only sources containing data relevant to anticancer therapeutic development or directly addressing clinical innovation in breast cancer were included. Additionally, reference lists of key publications were screened to identify materials not captured during the primary search.

The collected data were synthesized descriptively and organized according to the major biological subtypes of breast cancer and the principal categories of innovation, including modulation of signaling pathways, targeted and endocrine therapies, immunotherapy, antibody–drug conjugates, DNA damage–based approaches, and contemporary forms of photodynamic therapy. Priority was given to assessing their clinical, translational, and technological relevance, as well as their impact on the evolution of treatment standards between 2015 and 2025.

## 3. Results

Between 2015 and 2025, more than 9450 breast cancer–related clinical trials were registered in ClinicalTrials.gov, approximately 70% of which were phase II–III interventional studies. The most notable increase in new trial registrations occurred after 2018, paralleling the rapid expansion of targeted therapies and immunotherapies [20]. There are currently 15,914 registered studies for breast cancer [21].

Targeted therapies dominated the research landscape, with trial designs increasingly aligned with molecularly defined tumor subtypes. The largest proportion of studies involved hormone receptor–positive/HER2−negative (HR+/HER2−) disease, particularly those assessing CDK4/6 inhibitors (PALOMA, MONALEESA, MONARCH) [22] and agents targeting the PI3K–AKT–mTOR pathway (SOLAR-1, CAPItello-291) [18]. In HER2-positive and HER2-low tumors, the most significant advancements were observed in the development of antibody–drug conjugates (ADCs), including trastuzumab deruxtecan and trastuzumab emtansine, both of which markedly improved outcomes across patient populations with heterogeneous levels of HER2 expression [14].

In TNBC, a major milestone was the integration of immunotherapy with PD-1/PD-L1 inhibitors (IMpassion130, KEYNOTE-355) [23], along with the clinical implementation of PARP inhibitors (OlympiAD, EMBRACA), which demonstrated meaningful benefit in patients carrying BRCA1/2 mutations [24].

Across the last decade, there has also been a substantial rise in studies focusing on early-stage disease, high-risk populations, and combination treatment strategies. Adaptive platform designs—such as I-SPY, NATALEE, and DESTINY—were increasingly utilized, allowing simultaneous assessment of multiple therapeutic interventions within molecularly stratified cohorts [25,26]. Another notable trend was the integration of translational endpoints and molecular biomarkers into eligibility criteria; since 2020, more than half of newly initiated breast cancer trials have incorporated tumor genomic profiling into patient selection [27].

Overall, the 2015–2025 decade is characterized not only by the expanding volume of clinical research but also by a fundamental thematic shift—from traditional trial structures toward biologically driven designs integrating molecular data, biomarker-based stratification, and multi-omics technologies. Together, these elements define the contemporary paradigm of precision medicine in breast cancer. Detailed results from key clinical trials for each major breast cancer subtype are presented in Section 3.1, Section 3.2, Section 3.3 and Section 3.4.

### 3.1. HR+/HER2− Breast Cancer (2015–2025)

#### 3.1.1. Introduction and Biological Significance

Hormone receptor–positive/HER2-negative (HR+/HER2−) breast cancer is the most common biological subtype, accounting for approximately 70% of all cases [28]. Its development and maintenance are closely linked to the activity of the estrogen receptor (ER) and the progesterone receptor (PR), which regulate the expression of numerous genes involved in proliferation, differentiation, and survival of mammary epithelial cells [29]. Activation of ER increases the expression of cyclin D1, initiating the formation of CDK4/6–cyclin D complexes. These complexes subsequently phosphorylate the retinoblastoma (RB) protein, releasing its inhibitory effect on the E2F transcription factor and enabling the G1–S phase transition of the cell cycle [30]. Deregulation of this mechanism results in excessive cellular proliferation and loss of cell-cycle control, representing one of the fundamental pathogenic processes in HR-positive breast cancer [31]. The HR+/HER2−subtype is typically characterized by slower disease progression and a strong dependence on hormonal signaling, which makes it particularly responsive to endocrine therapy. Over time, however, endocrine resistance may emerge, driven by mechanisms such as ESR1 mutations, activation of the PI3K/AKT/mTOR pathway, or other adaptive cellular processes [32,33]. The intricate interplay between hormonal, proliferative, and molecular signaling pathways positions HR+/HER2− breast cancer as a quintessential example of a tumor in which underlying biological processes directly dictate therapeutic responsiveness.

#### 3.1.2. The Era of CDK4/6 Inhibitors

**Figure 1:**
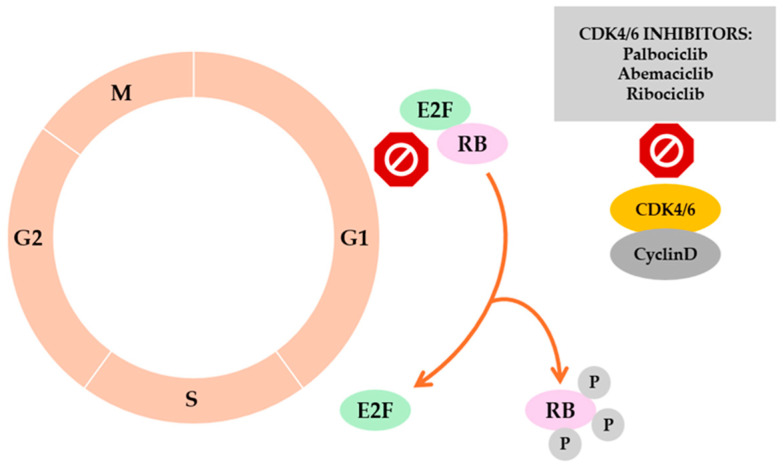
Schematic representation of cell-cycle blockade induced by cyclin-dependent kinase 4/6 (CDK4/6) inhibitors. CDK4/6–cyclin D–mediated phosphorylation of retinoblastoma protein (RB) leads to E2F transcription factor release and G1–S phase transition. CDK4/6 inhibition prevents RB phosphorylation, thereby maintaining E2F sequestration and inducing G1 cell-cycle arrest. G1, S, G2, and M denote cell-cycle phases; P indicates phosphorylation.

Dysregulation of cell-cycle control represents a fundamental hallmark of luminal HR+/HER2− breast cancer. As illustrated in Figure 1, overexpression of cyclin D1 (CCND1), amplification of CDK4, and loss of RB1 function are observed in a substantial proportion of cases and lead to uncontrolled phosphorylation of the RB–E2F complex, enabling G1–S transition in the absence of external proliferative cues [30,34,35]. Molecular studies have confirmed that activation of the ER–cyclin D–CDK4/6–RB axis constitutes a central proliferative mechanism in hormone-dependent breast cancer [36]. Throughout the early 2000s, the concept of pharmacologically targeting cyclin-dependent kinases was explored repeatedly; however, first-generation CDK inhibitors (e.g., flavopiridol, roscovitine) were limited by poor specificity and considerable toxicity [37]. The breakthrough came with the development of selective CDK4/6 inhibitors—enzymes directly responsible for RB phosphorylation. These agents (palbociclib, ribociclib, abemaciclib) demonstrated the ability to induce a reversible G1 cell-cycle arrest without causing DNA damage or eliciting permanent cytotoxicity [38].

Preclinical in vitro and in vivo models have demonstrated that CDK4/6 inhibitors act synergistically with endocrine therapy by restoring proliferative control in ER-positive cells, in which cyclin D1 expression is estrogen-induced [31,39]. In addition, these agents have been shown to induce features of cellular senescence, reduce translational activity, and promote immunomodulatory changes within the tumor microenvironment that facilitate T-cell recruitment [40]. These findings provided the biological rationale for launching extensive clinical development programs evaluating CDK4/6 inhibition in HR+/HER2− breast cancer, ultimately transforming the therapeutic paradigm for this disease. The first large, randomized, phase III clinical trials confirmed that blockade of the CDK4/6–RB axis combined with endocrine therapy leads to a substantial improvement in progression-free survival (PFS) and, over time, overall survival (OS). The most comprehensive dataset originates from the MONALEESA program, comprising three registrational studies—MONALEESA-2, MONALEESA-3, and MONALEESA-7. Each trial focused on a distinct clinical population, namely postmenopausal women, premenopausal patients, and individuals previously treated with endocrine therapy, respectively, thereby enabling a broad evaluation of ribociclib efficacy across clinically relevant settings. In the MONALEESA-2 study [41], 668 postmenopausal women with HR+/HER2− advanced breast cancer who had not received prior systemic therapy for advanced disease were enrolled. The addition of ribociclib to letrozole significantly prolonged median PFS to 25.3 months compared with 16.0 months in the placebo group (HR 0.56; 95% CI 0.43–0.72; *p* < 0.0001). The objective response rate (ORR) was 52.7% with ribociclib versus 37.1% with placebo. The safety profile was predictable, with hematologic adverse events being the most common, including neutropenia (59%) and leukopenia (21%). Grade ≥ 3 adverse events occurred in 21% of patients, and no treatment-related deaths were reported. A matching-adjusted indirect comparison (MAIC) published in 2023 demonstrated the superiority of ribociclib over palbociclib in terms of OS (HR 0.68; 95% CI 0.48–0.96), with comparable PFS outcomes [42]. Although this analysis does not constitute a direct head-to-head comparison, it supports the robust efficacy of ribociclib as a first-line treatment for postmenopausal patients. The MONALEESA-7 trial [43] was the first randomized phase III study to enroll premenopausal and perimenopausal women. All participants received pharmacologic ovarian suppression with goserelin, followed by endocrine therapy (tamoxifen or an aromatase inhibitor) combined with ribociclib or placebo. The results demonstrated a significant improvement in PFS, reaching 23.8 months in the ribociclib arm compared with 13.0 months in the placebo arm (HR 0.55; 95% CI 0.44–0.69; *p* < 0.0001). In the updated analysis, with a median follow-up of 53.5 months, a sustained overall survival (OS) benefit was confirmed—median OS was 58.7 months with ribociclib versus 48.0 months with placebo (HR 0.76; 95% CI 0.61–0.96) [44]. The four-year survival rate was 60% and 50%, respectively. The therapeutic effect was consistent across all examined subgroups, including patients younger than 40 years and those receiving aromatase inhibitors. This trial unequivocally established the clinical value of combining endocrine therapy (ET) with CDK4/6 inhibition in premenopausal women—a population historically considered more challenging to treat. The third pillar of the program, MONALEESA-3 [45], included postmenopausal women and men treated with fulvestrant in the first- or second-line setting. The addition of ribociclib prolonged median PFS to 20.5 months compared with 12.8 months in the placebo arm (HR 0.59; 95% CI 0.48–0.73; *p* < 0.001). In the most recent analysis from 2023 [46], median OS in the first-line subgroup reached 67.6 months versus 51.8 months in the control group (HR 0.67; 95% CI 0.50–0.90). These results further solidified the sustained overall survival advantage conferred by CDK4/6 inhibitors and cemented their role as a standard-of-care backbone in combination with either aromatase inhibitors or fulvestrant. The findings from registrational trials have been validated by real-world evidence. The phase IIIb CompLEEment-1 study [47], which included more than 3200 patients—among them men and individuals with ECOG ≤ 2 or stable CNS metastases—reported a median time to progression of 27.1 months and a clinical benefit rate (CBR) of approximately 78%. The safety profile was consistent with previous randomized trials, supporting the generalizability of CDK4/6 inhibition to routine clinical practice. In 2024, results from the RIGHT Choice trial [48] were published, comparing ribociclib plus ET with combination chemotherapy in premenopausal and perimenopausal women with clinically aggressive HR+/HER2− advanced breast cancer, including 68% with visceral metastases. The ribociclib + aromatase inhibitor (AI) + goserelin regimen achieved significantly longer PFS (21.8 vs. 12.8 months; HR 0.61; 95% CI 0.43–0.87; *p* = 0.003) and a comparable response rate (66.1% vs. 61.8%), while demonstrating lower systemic toxicity. Notably, although the time to first response was shorter in the chemotherapy arm (3.2 vs. 4.9 months), ET + CDK4/6 inhibition provided superior durability of benefit and improved quality of life. This study decisively challenged the long-standing assumption that patients experiencing so-called “visceral crisis” require immediate cytotoxic therapy, showing instead that CDK4/6-based regimens may represent a more effective and better-tolerated alternative. A somewhat different outcome was observed in the PEARL trial [49], which compared palbociclib combined with endocrine therapy versus capecitabine in postmenopausal women with aromatase inhibitor–resistant disease. In this population, palbociclib did not demonstrate superiority over chemotherapy in terms of PFS (7.5 vs. 10.0 months; HR 1.13; *p* = 0.32) or OS (34.0 vs. 29.2 months). Although these findings indicate that chemotherapy remains a viable option in cases of resistance to prior endocrine therapy, it is important to note that CDK4/6 inhibition was associated with a more favorable tolerability profile and improved quality of life. A further step toward treatment optimization involved assessing dose-dependent differences in efficacy and safety. The AMALEE study [50] compared ribociclib at 400 mg versus 600 mg in combination with aromatase inhibitors. Non-inferiority in ORR (47.2% vs. 54.4%) was not demonstrated; however, median PFS and duration of response (DOR) were comparable (26.9 vs. 25.1 months and 26.5 vs. 28.8 months, respectively). The 400 mg dose was associated with lower pharmacokinetic exposure (Cmax—28%, AUC—43%) and markedly reduced rates of grade 3–4 neutropenia (41% vs. 58.5%) and QTc prolongation (12.5 vs. 19.7 ms). In clinical practice, these findings support dose reduction to 400 mg in patients experiencing hematologic toxicity or QTc prolongation without compromising PFS efficacy. CDK4/6 inhibitors represent one of the most successful examples of biologically rational drug development translated into routine oncology practice. Across multiple randomized phase III trials, these agents consistently demonstrated substantial improvements in progression-free and overall survival, reshaping the standard-of-care for HR+/HER2− advanced breast cancer. Importantly, their favorable tolerability profiles enabled replacement of chemotherapy even in patients with visceral disease, redefining treatment sequencing. However, despite their clinical success, intrinsic and acquired resistance remains universal, underscoring the need for biomarker-guided sequencing and rational combination strategies. Thus, CDK4/6 inhibitors illustrate both the power and the limitations of targeted monotherapy in biologically adaptive tumors.

In summary, completed clinical studies clearly establish CDK4/6 inhibitors combined with endocrine therapy as the cornerstone of treatment for advanced HR+/HER2− breast cancer, regardless of age or menopausal status. Ribociclib has demonstrated the greatest consistency of outcomes across diverse patient populations, with a well-documented overall survival benefit. CDK4/6-based regimens can effectively replace chemotherapy even in patients with substantial visceral disease burden, offering superior tolerability and quality of life. The introduction of reduced-dose regimens (400 mg) further expands opportunities for individualized therapy without loss of efficacy. A detailed summary of the key completed clinical trials evaluating CDK4/6 inhibitors in HR+/HER2− breast cancer is provided in Table 1.

Following the demonstration of efficacy of CDK4/6 inhibitors in advanced HR+/HER2− breast cancer, the next phase of clinical development involved evaluating their role in early-stage disease. One of the largest studies of this type was the PALLAS trial (NCT02513394), initiated in 2015. More than 5700 patients with early HR+/HER2− breast cancer who had undergone surgical treatment were enrolled and received palbociclib (125 mg orally, 21/28 days for 2 years) in combination with standard endocrine therapy, or endocrine therapy alone. Results published in 2022 showed no benefit from adding palbociclib to adjuvant treatment—the 4-year invasive disease-free survival (iDFS) rates were 84.2% vs. 84.5% in the control arm [51]. A high discontinuation rate due to adverse events (27%) additionally underscored the limited tolerability of long-term treatment in this population. The 2025 update likewise demonstrated no advantage in any analyzed subgroup [52], suggesting that the efficacy of CDK4/6 inhibition in the setting of minimal residual disease may be limited. Despite its negative outcome, the PALLAS trial provided important biological and clinical insights and represents a key reference point for subsequent adjuvant studies.

Beyond completed and ongoing trials, the study period also included several early-terminated projects closed for organizational or methodological reasons rather than safety signals or lack of efficacy. Although these trials did not yield mature clinical data, they highlight the practical challenges associated with conducting large-scale translational and therapeutic studies in patients with HR+/HER2− breast cancer.

One such project was the companion study to CompLEEment-1 (NCT03050398) conducted by Novartis. This non-interventional trial aimed to collect biological material from patients with advanced HR+/HER2− breast cancer treated with ribociclib to identify molecular mechanisms of resistance to CDK4/6 inhibition. Tissue samples were planned at two time points—at baseline and at disease progression. However, the study was terminated prematurely after enrolling only eight patients due to procedural irregularities constituting violations of Good Clinical Practice (GCP), as well as the closure of the parent CompLEEment-1 trial, which halted further recruitment. No adverse events or safety concerns were identified, and the collected samples were not analyzed. Although the project did not produce actionable clinical data, it underscored the critical importance of rigorous quality control and adherence to GCP standards in biomarker-driven studies accompanying large clinical trials [NCT03050398].

Another example of an early-terminated study is NCT04031885, conducted by Eli Lilly. This was a randomized, open-label, phase IV trial comparing the efficacy of abemaciclib (LY2835219) in combination with fulvestrant versus investigator’s choice chemotherapy in women with HR+/HER2− metastatic breast cancer with visceral involvement. The study aimed to determine whether a CDK4/6 inhibitor could serve as an effective and better-tolerated alternative to cytotoxic therapy in a population characterized by particularly aggressive disease biology.

Despite the clinical relevance of the research question, the trial was discontinued prematurely for sponsor-related strategic reasons, with no reported issues concerning safety or efficacy. Results were not published, and the absence of PFS and OS data precludes any assessment of a potential advantage of abemaciclib over chemotherapy in this patient population [NCT04031885].

While ribociclib has the most extensive phase III evidence base and demonstrated OS benefit across multiple trials, palbociclib and abemaciclib also represent important options within the CDK4/6 inhibitor class. Palbociclib (PALOMA program) has shown PFS benefit in both first-line (PALOMA-2) and endocrine-resistant (PALOMA-3) settings, although adjuvant studies (PALLAS) did not demonstrate benefit. Abemaciclib (MONARCH program) features continuous dosing, greater CDK4 selectivity, and a distinctive safety profile characterized by higher rates of diarrhea and lower rates of neutropenia. Inclusion of these agents highlights the overall consistency of the CDK4/6 inhibitor class while acknowledging differences in administration, toxicity, and evidence base. In summary, completed clinical studies clearly establish CDK4/6 inhibitors combined with endocrine therapy as the cornerstone of treatment for advanced HR+/HER2− breast cancer, regardless of age or menopausal status. Ribociclib has demonstrated the greatest consistency of outcomes across diverse patient populations, with a well-documented overall survival benefit. CDK4/6-based regimens can effectively replace chemotherapy even in patients with substantial visceral disease burden, offering superior tolerability and quality of life.

#### 3.1.3. PI3K/AKT/mTOR Pathway Inhibitors

**Figure 2:**
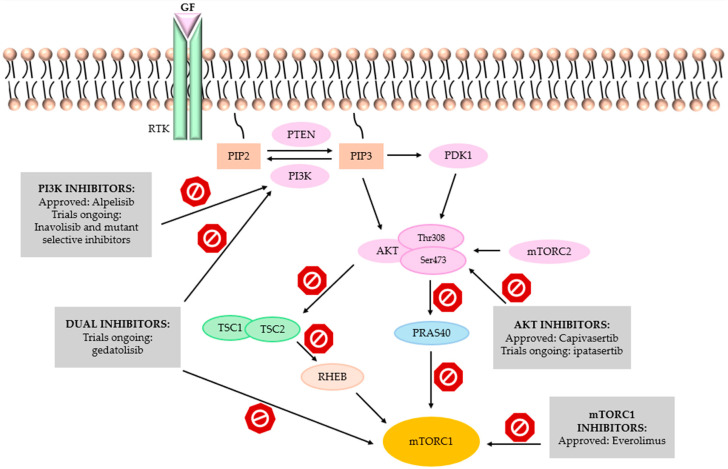
The phosphatidylinositol 3-kinase (PI3K)/protein kinase B (AKT)/mechanistic target of rapamycin (mTOR) signaling pathway and therapeutic points of intervention. Growth factor (GF)–mediated activation of receptor tyrosine kinases (RTKs) leads to PI3K-dependent conversion of phosphatidylinositol-4,5-bisphosphate (PIP2) to phosphatidylinositol-3,4,5-trisphosphate (PIP3), which activates AKT via phosphoinositide-dependent kinase-1 (PDK1) and mTOR complex 2 (mTORC2). AKT regulates downstream targets including tuberous sclerosis complex 1/2 (TSC1/2), Ras homolog enriched in brain (RHEB), and proline-rich AKT substrate 40 kDa (PRAS40), leading to activation of mTOR complex 1 (mTORC1). PTEN denotes phosphatase and tensin homolog. Red symbols indicate pharmacological points of inhibition.

As illustrated in Figure 2, the PI3K/AKT/mTOR signaling pathway is a central regulator of cellular proliferation, survival, and metabolic activity, and its deregulation represents one of the most frequent molecular events in HR+/HER2− breast cancer [53]. Activation of receptor tyrosine kinases leads to the conversion of PIP2 into PIP3 and the recruitment of AKT, whose phosphorylation at Thr308 and Ser473 initiates an effector cascade resulting in inhibition of the TSC1/TSC2 complex, activation of RHEB, and stimulation of mTORC1 [54]. The mTORC1 complex enhances translational activity through phosphorylation of 4EBP1 and S6K, facilitating G1–S cell-cycle progression and promoting tumor growth [55].

The most commonly observed activating aberrations in HR+/HER2− disease are *PIK3CA* mutations, present in approximately 35–45% of patients [56]. These alterations lead to elevated PIP3 levels and sustained AKT activation. Another key mechanism is loss of PTEN function, whose physiological role is to suppress signaling by converting PIP3 back to PIP2 [57]. Less frequently, activating mutations in *AKT1* (e.g., E17K) are identified, stabilizing the kinase in an active conformation [58]. Collectively, these abnormalities drive proliferation, suppress apoptosis, and reprogram cellular metabolism, enabling tumor cells to survive despite endocrine therapy.

Hyperactivation of the PI3K/AKT/mTOR axis is also among the best-characterized mechanisms of endocrine resistance, as AKT and mTORC1 potentiate estrogen receptor signaling via both genomic and non-genomic pathways [59]. Preclinical studies have demonstrated that pharmacologic inhibition of this pathway restores sensitivity to aromatase inhibitors and SERDs and reduces translational and metabolic activity in tumor cells [60].

Advances in molecular biology have driven the rapid development of targeted therapies directed at the PI3K/AKT/mTOR pathway. The first agent to receive regulatory approval was alpelisib, a selective PI3Kα inhibitor designated for patients with PIK3CA-mutated disease [18]. Subsequent years provided convincing evidence supporting inhibition of additional pathway components. AKT inhibitors—led by capivasertib—demonstrate activity irrespective of the underlying molecular aberration (PIK3CA, PTEN, AKT1) [61], while everolimus, an mTORC1 inhibitor, reduces the translational and metabolic consequences of pathway hyperactivation [62]. In parallel, dual PI3K/mTOR inhibitors such as gedatolisib are being evaluated [63]. Suppression of the PI3K/AKT/mTOR axis leads to reduced cellular proliferation, downregulation of pro-proliferative proteins, and, clinically, to prolonged disease control.

The efficacy of this therapeutic class has been confirmed across completed randomized clinical trials, although the developmental trajectory of individual agents varied substantially. Early pan-PI3K inhibitors—such as pictilisib—failed to achieve meaningful clinical benefit. In the FERGI trial [64], adding pictilisib to fulvestrant in postmenopausal women with AI-resistant HR+/HER2− advanced breast cancer did not significantly improve PFS, either in the overall population (6.6 vs. 5.1 months; HR 0.74; *p* = 0.096) or in the *PIK3CA*-mutant subgroup (5.4 vs. 10.0 months; HR 1.07; *p* = 0.84). At the same time, toxicity was substantial (≥G3 in 36–61% of patients), leading to discontinuation of clinical development. These results underscored the need for greater selectivity and precise patient stratification.

A major breakthrough came with selective PI3Kα inhibition. In the SOLAR-1 trial [18], which enrolled 572 patients who had progressed on AI therapy, the addition of alpelisib to fulvestrant more than doubled median PFS in *PIK3CA*-mutant tumors (11.0 vs. 5.7 months; HR 0.65; *p* < 0.001), accompanied by higher objective response rates. The lack of benefit in *PIK3CA*-wild-type tumors confirmed the necessity of molecular selection. The most common adverse events reflected on-target PI3Kα inhibition—hyperglycemia, rash, and diarrhea—leading to treatment discontinuation in approximately 25% of patients. Despite this, SOLAR-1 served as the registrational trial and enabled broad clinical implementation of alpelisib.

The activity of alpelisib was further supported in the setting of CDK4/6 inhibitor resistance. In the prospective BYLieve trial [65], clinically meaningful disease control was achieved in patients progressing after CDK4/6 + AI or fulvestrant therapy, with median PFS of 5.6–12.0 months depending on the cohort. More than 50% of patients remained progression-free at 6 months, and durable stabilization was observed in 27–30%. The safety profile was predictable and consistent with SOLAR-1, confirming the value of PI3Kα inhibition after first-line treatment failure.

In parallel, agents targeting other nodes of the PAM pathway were evaluated. Inhibition of insulin-like growth factor signaling (IGF-1/2), capable of activating AKT independently of PI3K, was the basis for the XENERA-1 trial [66]. Adding xentuzumab to everolimus + exemestane in patients with non-visceral disease did not improve PFS compared with placebo, either in independent review (12.7 vs. 11.0 months; HR 1.19; *p* = 0.65) or investigator assessment (7.4 vs. 9.2 months; HR 1.23). Although the regimen was well tolerated, the absence of clinical benefit led to discontinuation of its development.

In summary, completed clinical trials clearly demonstrate that the effectiveness of PI3K-directed therapy depends strongly on molecular selectivity and appropriate patient selection. A detailed comparison of populations, treatment regimens, and efficacy and safety outcomes from completed trials of PI3K/AKT/mTOR inhibitors is presented in Table 2.

The next stage in the development of therapies targeting the PI3K/AKT/mTOR pathway involves the evaluation of next-generation inhibitors and combination strategies designed to achieve multilayered blockade of proliferative signaling. Particular attention has been directed toward selective PI3Kα inhibitors, broad-spectrum AKT inhibitors, and dual PI3K/mTOR inhibitors. Emerging data from indirect comparative analyses suggest that their greatest clinical potential may lie in overcoming endocrine resistance following progression on CDK4/6 inhibitor–based therapy, which currently represents the predominant mechanism of treatment failure in patients with advanced HR+/HER2− breast cancer.

The most advanced clinical program in this field is the INAVO120 trial (NCT04191499) [67], a randomized phase II/III study evaluating the selective PI3Kα inhibitor inavolisib in combination with palbociclib and fulvestrant in patients with PIK3CA-mutated disease. The study enrolled individuals with locally advanced or metastatic breast cancer who experienced recurrence during, or within 12 months after completing, adjuvant endocrine therapy—corresponding to the classical definition of primary endocrine resistance. The primary analysis, published in 2024, demonstrated a significant improvement in PFS, reaching 15.0 months compared with 7.3 months in the control arm (HR 0.43; 95% CI 0.32–0.59; *p* < 0.001), along with more than a two-fold increase in objective response rates. The final overall survival analysis (2025) [68] confirmed the clinical relevance of this benefit, showing a median OS of 34.0 versus 27.0 months (HR 0.67; *p* = 0.02), marking the first unequivocal demonstration that modular PI3Kα blockade can improve OS after progression on CDK4/6 inhibitor–based therapy. The safety profile of inavolisib was characterized primarily by hematologic toxicities, moderate rates of hyperglycemia and ocular events, and a low frequency of permanent treatment discontinuation (6.8%). Collectively, these findings suggest that adding a selective PI3Kα inhibitor to CDK4/6 + SERD therapy may become a new standard second-line option for PIK3CA-mutant HR+/HER2− disease.

A second pivotal study in this domain is CAPItello-291 (NCT04305496) [69], which assessed the AKT inhibitor capivasertib in combination with fulvestrant in patients with locally advanced or metastatic disease following progression during or after treatment with an aromatase inhibitor. In contrast to PI3Kα-selective inhibitors, capivasertib suppresses downstream signaling independently of the underlying genomic aberration (PIK3CA, AKT1, PTEN), thereby broadening the population of potential responders. The 2023 analysis demonstrated a significant improvement in PFS both in the overall population and in patients with PAM-pathway alterations. Quality-of-life outcomes published in 2024 indicated that treatment did not worsen global functioning, with transient diarrhea representing the most frequent adverse event. These results further underscore that AKT inhibitors may offer meaningful therapeutic benefit even in patients without a precisely defined PIK3CA mutation, distinguishing them conceptually from therapies based on selective PI3Kα blockade.

Further confirmation of the clinical activity of AKT inhibitors was provided by the FINER/MA.40 trial (NCT04650581) [70], which evaluated ipatasertib in combination with fulvestrant following failure of first-line therapy consisting of a CDK4/6 inhibitor plus an aromatase inhibitor. The median PFS was 5.32 versus 1.94 months (HR 0.61; *p* = 0.0007), with an even more pronounced effect in the subgroup harboring PIK3CA/AKT1/PTEN alterations (HR 0.47). The toxicity profile was dominated by diarrhea and fatigue, whereas the rate of permanent treatment discontinuation remained low (6.5%). The FINER study underscores that pharmacologic AKT inhibition can effectively overcome secondary resistance to endocrine therapy and CDK4/6 inhibition, which is of substantial clinical relevance.

In parallel, agents with broader mechanisms of action—capable of simultaneous PI3K and mTOR blockade—are being investigated. The most advanced program in this category is the phase III VIKTORIA-1 trial (NCT05501886), which evaluates gedatolisib combined with fulvestrant, with or without palbociclib, in patients with HR+/HER2− breast cancer who progressed on CDK4/6 inhibitor + AI therapy. The experimental regimen is being compared with standard-of-care options, including fulvestrant and alpelisib for PIK3CA-mutant disease. Given that gedatolisib inhibits both PI3K and mTOR, it may mitigate adaptive bypass mechanisms—one of the principal causes of secondary resistance to selective PI3K inhibitors. Recruitment has been completed, and molecular and clinical analyses are ongoing. The outcome of this development program may define the role of dual PI3K/mTOR blockade in the therapeutic sequence for endocrine-resistant HR+/HER2− advanced breast cancer.

During the analyzed period, several trials were terminated before full clinical results were obtained. Although they did not yield complete datasets, they provided important insights into safety, feasibility, and therapeutic limitations within the PI3K/AKT/mTOR axis.

The most advanced among these was the phase III BELLE-3 trial (NCT01633060) [71], which evaluated the pan-PI3K inhibitor buparlisib in combination with fulvestrant in patients with HR+/HER2− advanced breast cancer previously treated with endocrine therapy and an mTOR inhibitor. Despite showing a significant improvement in PFS (3.9 vs. 1.8 months; HR 0.67; *p* = 0.0003), the regimen was associated with substantial toxicity—particularly grade 3–4 hepatotoxicity and hyperglycemia—which led to frequent treatment discontinuations. Consequently, the sponsor decided to halt the buparlisib development program. These results highlighted the limitations of pan-PI3K inhibition and directly stimulated the development of selective PI3Kα inhibitors, such as alpelisib, with more favorable safety profiles.

Another prematurely terminated project was NCT05966584, which investigated prophylactic strategies for alpelisib-associated rash at treatment initiation. The trial was stopped due to insufficient recruitment, without methodological concerns or safety issues, reflecting the practical challenges of enrolling highly selected populations. A similar outcome occurred in the pilot decentralized TELEPIK study (NCT04862143), which evaluated alpelisib plus fulvestrant under a telemedicine-supervised model. The study was discontinued due to low accrual, illustrating recruitment barriers inherent to remote clinical designs and biomarker-defined populations.

The development program for the PI3K inhibitor taselisib was also terminated following the phase III SANDPIPER trial (NCT02340221) [72]. Although evidence of antitumor activity was observed, the overall clinical benefit was modest, and toxicity exceeded acceptable thresholds considering available therapeutic alternatives. The discontinuation was strategic in nature and was not related to procedural safety concerns. Targeting the PI3K/AKT/mTOR axis has yielded mixed translational outcomes, highlighting the importance of molecular selectivity and patient stratification. Early-generation pan-PI3K inhibitors demonstrated limited clinical utility due to unacceptable toxicity, effectively halting their development. In contrast, isoform-selective PI3Kα inhibitors and downstream AKT inhibitors successfully translated molecular insights into clinically actionable therapies, particularly in biomarker-defined populations. These findings emphasize that pathway inhibition is clinically meaningful only when aligned with tumor genomics, reinforcing precision oncology principles. Nonetheless, metabolic toxicity and limited durability of response remain key challenges, positioning PI3K/AKT inhibition primarily as a second-line strategy following CDK4/6 inhibitor failure.

#### 3.1.4. New SERDs, ESR1 Mutations, and Biomarkers

Selective estrogen receptor modulators (SERMs) and selective estrogen receptor degraders (SERDs) constitute key classes of therapies targeting estrogen receptor α (ERα), the dominant driver of proliferation in hormone receptor–positive, HER2-negative breast cancer. SERMs, represented by agents such as tamoxifen and raloxifene, competitively bind to the ligand-binding domain of ER, inducing a conformational shift that prevents the recruitment of co-activators essential for the transcriptional activation of estrogen-responsive genes. Although SERMs effectively inhibit ER-mediated transcription, the receptor remains present within the cell, allowing potential reactivation through alternative growth pathways or microenvironment-driven signaling [72].

**Figure 3:**
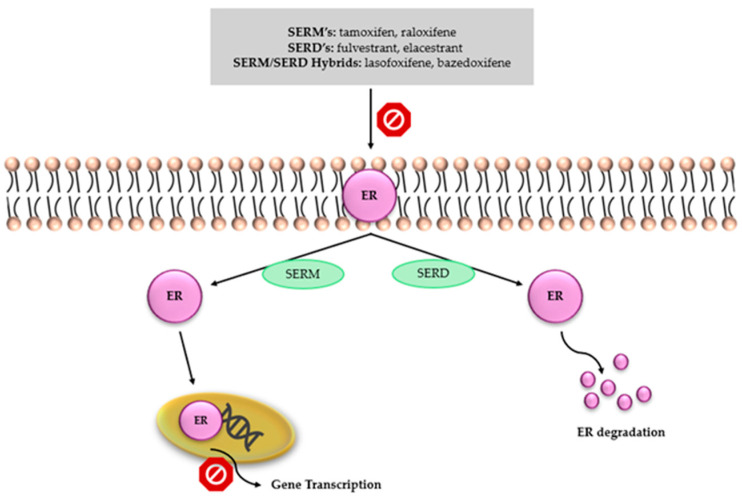
Mechanism of action of selective estrogen receptor modulators (SERMs) and selective estrogen receptor degraders (SERDs). SERMs (e.g., tamoxifen, raloxifene) bind to estrogen receptor (ER) and modulate its activity, inhibiting gene transcription. SERDs (e.g., fulvestrant, elacestrant) bind to ER and induce its degradation. Hybrid compounds (e.g., lasofoxifene, bazedoxifene) exhibit combined SERM and SERD activities.

SERDs operate via a distinct mechanism—upon binding to ER, they stabilize a receptor conformation that promotes ubiquitination and proteasomal degradation, leading to sustained loss of ER protein. This results in the elimination of both ligand-dependent and ligand-independent ER signaling, a feature of particular importance in the context of resistance to aromatase inhibitors and SERMs [73]. ER degradation suppresses the expression of proliferative genes, decreases cyclin D1 levels, and induces G1 cell-cycle arrest, while also reducing the selective pressure that promotes clonal evolution and therapeutic resistance [74]. A schematic representation of the mechanisms of action of SERMs and SERDs is shown in Figure 3.

Somatic mutations in the *ESR1* gene have emerged as a critical biological driver increasingly identified in patients with metastatic disease previously exposed to prolonged aromatase inhibitor–induced estrogen deprivation. The most frequent ligand-binding domain mutations, including Y537S, Y537N, and D538G, induce a receptor conformation that remains constitutively active in the absence of estrogens, thereby sustaining proliferative signaling despite optimal endocrine therapy [75,76]. This mechanism represents one of the principal molecular bases of resistance to aromatase inhibitors. Because SERDs degrade both wild-type and mutant ER, they offer a potentially more effective therapeutic strategy for patients harboring *ESR1* mutations [77].

The ability to detect these mutations in circulating tumor DNA (ctDNA) has enabled real-time monitoring of clonal evolution under treatment pressure and opened the path toward adaptive therapeutic strategies in which a switch in endocrine therapy is implemented at the moment of molecular detection of resistance—prior to radiological progression [78]. The integration of ER degradation with liquid biopsy–based monitoring represents one of the most significant advances in endocrine therapy development between 2021 and 2025.

The most mature clinical data derive from the EMERALD trial, a randomized phase III study comparing elacestrant with standard endocrine monotherapy (fulvestrant or an aromatase inhibitor) in patients with ER+/HER2− breast cancer previously treated with a CDK4/6 inhibitor. Elacestrant significantly improved progression-free survival in the overall population and—more prominently—in patients with *ESR1* mutations. The benefit was particularly pronounced in patients who had achieved a prolonged response to prior CDK4/6 inhibitor–based therapy, likely reflecting persistent tumor dependence on ER signaling and the clonal selection of AI-resistant subpopulations. Elacestrant demonstrated a manageable safety profile, with nausea—typically mild—representing the most common adverse event. These results led to the regulatory approval of elacestrant as the first oral SERD with proven superiority over endocrine monotherapy [79].

A new generation of SERDs is being developed to support personalized therapy and allow earlier molecular intervention. The most groundbreaking example is camizestrant, evaluated in the SERENA-6 trial, in which patients receiving first-line therapy with an aromatase inhibitor plus a CDK4/6 inhibitor underwent serial monitoring for *ESR1* mutations in ctDNA. Upon detection of a mutation—prior to radiographic progression—patients were switched to camizestrant while continuing the same CDK4/6 inhibitor. This strategy extended median PFS by more than six months compared with continued aromatase inhibitor therapy. These findings provide the first clinical evidence that dynamic ctDNA monitoring can serve as a predictive biomarker for impending endocrine resistance and enable a pre-emptive therapeutic switch to overcome emerging resistance. Treatment tolerability remained favorable, with minimal discontinuation due to toxicity [80].

In parallel, imlunestrant is being evaluated as an orally bioavailable SERD with central nervous system penetration and full antagonistic activity against ER. In the EMBER-3 trial, imlunestrant improved PFS in the *ESR1*-mutated population; however, no superiority over standard endocrine therapy was observed in the overall cohort. In combination studies, the addition of imlunestrant to abemaciclib prolonged disease control but was associated with increased toxicity, predominantly hematologic and gastrointestinal. These findings suggest that the therapeutic benefit of oral SERDs is greatest in patients with confirmed *ESR1* mutations, whereas combination strategies may be appropriate for individuals with a more aggressive disease phenotype [81].

Subsequent clinical programs further demonstrate that ER modulation remains effective even in clinical scenarios where aromatase inhibitors are poorly tolerated or contraindicated. An example of this strategy is the PATHWAY trial, in which tamoxifen combined with palbociclib significantly prolonged PFS compared with tamoxifen alone, with a toxicity profile dominated by hematologic adverse events typical for CDK4/6 inhibitors but without a meaningful increase in infectious complications. Similarly, early data from the combination of dalpiciclib with toremifene indicate the potential of SERM-based therapy administered together with CDK4/6 inhibitors, particularly in patients intolerant to aromatase inhibitors [82].

At the same time, several next-generation oral SERDs are in development, including palazestrant (OP-1250), currently under evaluation in the ongoing OPERA-01 trial, which compares the agent with standard endocrine monotherapy in patients previously treated with CDK4/6 inhibitors. As a global study, its results will be crucial in determining whether emerging oral degraders can surpass the efficacy demonstrated by elacestrant and imlunestrant.

Not all clinical programs have been successful. The AMEERA-6 trial, which evaluated amcenestrant in early breast cancer, was terminated early at the sponsor’s discretion, despite the absence of safety concerns. The decision reflected the overall lack of efficacy observed for amcenestrant in other studies, underscoring that the development of oral SERDs requires rigorous molecular selection and stringent clinical validation.

Although the number of phase III trials investigating oral SERDs remains smaller compared with anti-HER2 therapies or CDK4/6 inhibitors, their clinical significance is disproportionately high. This is largely because these studies target biologically selected populations—particularly patients with *ESR1* mutations or resistance induced by prior endocrine therapy. Consequently, each subsequent study, even single-center or early-phase, provides meaningful evidence. The breakthroughs observed in EMERALD and SERENA-6—namely, the efficacy of oral SERDs after loss of response to AI and CDK4/6 inhibitors, as well as the feasibility of “anticipatory” treatment changes guided by ctDNA—are redefining therapeutic strategies for HR+/HER2− breast cancer. In the coming years, indications are expected to expand, and new molecules will emerge; however, the direction of development is clear: real-time monitoring of molecular evolution and targeted overcoming of endocrine resistance.

The emergence of oral SERDs and real-time ESR1 mutation monitoring represents a paradigm shift in endocrine therapy. Unlike earlier endocrine agents, these strategies directly address molecular mechanisms of resistance rather than merely suppressing estrogen production. The success of elacestrant and ctDNA-guided treatment adaptation in SERENA-6 provides the first clinical proof that anticipatory intervention based on molecular evolution can delay disease progression. However, benefits are largely confined to genomically selected populations, and combination strategies may be required for broader applicability. Collectively, these developments signal a transition from static endocrine sequencing toward dynamic, biomarker-driven disease management.

#### 3.1.5. PROTAC-Mediated Estrogen Receptor Degradation as a Novel Endocrine Strategy

Beyond receptor antagonism and conventional selective estrogen receptor degradation, targeted protein degradation has emerged as a next-generation therapeutic concept in hormone receptor–positive breast cancer, aiming to eliminate the estrogen receptor itself and thereby overcome adaptive mechanisms of endocrine resistance. ARV-471 (PF-07850327) is an orally bioavailable proteolysis-targeting chimera (PROTAC) designed to selectively degrade the estrogen receptor, representing a mechanistically distinct approach compared with conventional SERDs. In the phase 3 VERITAC-2 trial, ARV-471 was evaluated versus fulvestrant in patients with ER-positive, HER2-negative advanced breast cancer whose disease had progressed following prior endocrine-based therapy. This study constitutes the first phase 3 evaluation of a PROTAC in this disease setting, and results presented at a recent international oncology meeting demonstrated encouraging clinical activity. Collectively, these findings highlight targeted protein degradation as a promising new direction in the evolving landscape of endocrine therapies for advanced breast cancer [83].

### 3.2. HER2+ Breast Cancer (2015–2025)

#### 3.2.1. Introduction and Biological Significance

HER2-positive (HER2+) breast cancer accounts for approximately 15–20% of all diagnoses and has historically represented one of the most aggressive clinical subtypes. It is characterized by a high-proliferation phenotype and pronounced mitotic drive, translating into an increased likelihood of early metastatic dissemination and a distinct predilection for central nervous system (CNS) involvement already at advanced stages of disease [84,85,86]. Although substantial therapeutic progress has been achieved through modulation of the HER2 axis with multiple drug classes (monoclonal antibodies, tyrosine kinase inhibitors, and ADCs), disease behavior continues to be strongly influenced by the biological heterogeneity of the tumor and its microenvironment [84,87].

From a pathobiological standpoint, the most relevant features of HER2+ disease include: (I) marked spatial and temporal variability of HER2 expression, generating pronounced intratumoral heterogeneity (ITH), which correlates with lower pathological complete response (pCR) rates and inferior outcomes following neoadjuvant therapy [88]; (II) frequent co-expression of steroid hormone receptors (HR+/HER2+), creating functional crosstalk between proliferative HER2 signaling and endocrine pathways, thereby promoting resistance through activation of the PI3K/AKT/mTOR axis and dysregulation of cell-cycle control [84,87]; and (III) the presence of non-canonical HER2 isoforms, such as Δ16HER2 or the proteolytically truncated p95HER2, which possess enhanced transforming potential, partially evade the effects of extracellular domain–targeted antibodies, and amplify downstream pro-proliferative signaling [89]. Mechanisms of resistance and disease progression in HER2+ cancer are multifactorial. Well-characterized contributors include activating PIK3CA mutations, which are associated with reduced pCR rates and diminished sensitivity to HER2 blockade, as well as loss of PTEN, a key negative regulator of PI3K signaling [90]. Additional escape routes involve compensatory activation of non-ERBB receptors and signaling hubs (e.g., IGF1R, FGFR), phenotypic switching toward epithelial–mesenchymal transition (EMT), and metabolic reprogramming [87,91,92]. The immune microenvironment also plays an essential role. The efficacy of anti-HER2 antibodies is partly mediated by antibody-dependent cellular cytotoxicity (ADCC), influenced by Fcγ receptor polymorphisms and the density of tumor-infiltrating lymphocytes (TILs) [86,88,93]. Alterations in antigen presentation and immune checkpoint expression can further facilitate immune evasion [93]. At the organ level, HER2+ tumors exhibit a strong tropism for the CNS, which remains clinically significant despite improvements in systemic disease control. Limited penetration of many anti-HER2 agents across the blood–brain barrier has therefore become a critical determinant of therapeutic success [85,86]. Finally, the advent of next-generation antibody–drug conjugates (ADCs) with bystander-killing properties has fundamentally altered the therapeutic landscape—not only for classically HER2-amplified tumors but also for subsets with lower expression levels. Their clinical efficacy depends on HER2 internalization dynamics, lysosomal processing, and the physicochemical properties of the cytotoxic payload [14,92]. Taken together, the HER2+ subtype remains a paradigm of a receptor-driven tumor in which clinical outcomes closely reflect the interplay between receptor signaling, tumor heterogeneity, and immune engagement [84,87].

HER2-positive breast cancer remains a model for receptor-driven oncogenesis in which therapeutic success closely mirrors advances in molecular engineering. While early HER2 blockade dramatically improved outcomes, contemporary progress increasingly depends on overcoming biological heterogeneity, CNS tropism, and immune escape. Innovations in antibody engineering and ADC design have extended therapeutic benefit beyond classical HER2 amplification, reshaping both disease classification and treatment paradigms.

Given that much of contemporary anti-HER2 innovation is driven by patent-protected technological development—rather than established clinical evidence—the following subsection focuses on an analysis of selected patent disclosures from 2015 to 2025.

#### 3.2.2. Antibody–Drug Conjugates (ADC)

In recent years, ADCs have emerged as a central direction in the development of targeted therapies for HER2-positive breast cancer. By combining the specificity of an anti-HER2 monoclonal antibody with a highly cytotoxic payload, ADCs enable selective elimination of tumor cells while limiting systemic toxicity. Unlike conventional antibodies, ADCs can exert a bystander effect, whereby the released cytotoxic agent diffuses into adjacent tumor cells with lower HER2 expression—an attribute of particular relevance in biologically heterogeneous tumors. The following examples are derived from patent disclosures and are presented to illustrate emerging technological concepts and innovation activity rather than clinically validated therapeutic efficacy.

One example of this technological advancement is described in patent application AU2025201016A1 [94], which presents an anti-HER2 ADC incorporating a cytotoxic payload (DX) linked through an innovative peptide-based “GGFG” linker. The proposed structure—(succinimid-3-yl-N)-CH_2_CH_2_CH_2_CH_2_CH_2_-C(=O)-GGFG-NH-CH_2_-O-CH_2_-C(=O)-(NH–DX)—allows precise intracellular drug release following ADC internalization, thereby enhancing tumor selectivity and minimizing off-target toxicity. This design represents a next-generation ADC with improved penetration of resistant subclones and increased efficacy in tumors characterized by heterogeneous HER2 expression.

A subsequent innovation step is reflected in WO2025164597A1 [95], which proposes combining ADCs with immunotherapy, particularly immune checkpoint inhibitors (PD-1/PD-L1, CTLA-4). The synergistic effect arises from direct tumor-cell killing by ADCs in parallel with immune reactivation mediated by checkpoint blockade, enabling the reversal of resistance mechanisms commonly observed in advanced HER2+ malignancies. This strategy aligns with the broader therapeutic trend of integrating targeted agents with immunomodulators to enhance treatment efficacy within immunosuppressive tumor microenvironments.

In contrast, patent JP7674542B2 [96] focuses on a technological advancement: an optimized manufacturing method for ADCs incorporating topoisomerase I inhibitor derivatives (e.g., exatecan). The improved purification process minimizes protein aggregation, thereby increasing conjugate stability and enhancing production scalability. This innovation provides a technological foundation for platforms such as trastuzumab deruxtecan (T-DXd), which has become a key component of modern HER2-targeted therapy.

Collectively, these patent disclosures illustrate the technological evolution of ADC platforms, encompassing linker chemistry, combination concepts, and manufacturing optimization. While these innovations highlight important development directions, their clinical relevance remains dependent on subsequent experimental and clinical validation. Detailed information regarding the individual patent filings—including publication year, ownership, nature of innovation, and protection status—is summarized in Table 3.

Next-generation ADCs have fundamentally altered the therapeutic landscape of HER2-positive and HER2-low breast cancer. Their capacity for bystander killing addresses intratumoral heterogeneity that limits conventional antibody efficacy. Importantly, ADC success is not solely antigen-dependent but critically influenced by linker stability, payload potency, and intracellular processing. While ADCs such as trastuzumab deruxtecan have already redefined standards of care, ongoing innovations primarily represent platform optimization rather than paradigm shifts, emphasizing incremental yet clinically meaningful progress.

#### 3.2.3. Anti-HER2 Monoclonal Antibodies

Monoclonal antibodies targeting the HER2 receptor have constituted the backbone of HER2-positive breast cancer therapy for more than two decades. In recent years, however, innovation in this field has increasingly focused on antibody engineering, formulation strategies, and delivery technologies, rather than on the discovery of entirely new HER2-binding epitopes. Current development efforts therefore aim not only to optimize biological activity against HER2 but also to improve pharmacokinetics, patient accessibility, and organ penetration—particularly across the blood–brain barrier (BBB). The following examples are derived from patent disclosures and are discussed to illustrate technological and formulation-oriented innovation rather than to provide independent evidence of clinical efficacy.

Patent US20250018032A1 [97] describes a patent-protected therapeutic application involving dual HER2 blockade with pertuzumab and trastuzumab in combination with chemotherapy in the adjuvant setting of early HER2-positive breast cancer. The disclosure outlines dosing strategies and treatment regimens intended to support intensified HER2 pathway inhibition in high-risk patients. While the clinical benefit of dual HER2 blockade in this setting has been established in Phase III trials, the patent itself primarily addresses aspects of therapeutic application and regimen optimization rather than generating new clinical outcome data. This approach reflects the broader strategy of early, sustained HER2 pathway suppression to reduce the risk of disease progression. In contrast, US20240269064A1 [98] focuses on pharmaceutical formulation innovation, presenting fixed-dose subcutaneous preparations of anti-HER2 antibodies, including pertuzumab and co-formulations with trastuzumab. The disclosed technology enables both antibodies to be administered via a single subcutaneous injection, aimed at reducing administration time and improving treatment convenience, while preserving antibody integrity and biological activity. This approach exemplifies a wider trend toward optimizing routes of administration for biologics, facilitating outpatient care and reducing the burden on infusion-based healthcare infrastructure. The most advanced delivery-oriented concept is represented by US20230212312A1/US12331134B2 [99], which describes trispecific antibodies capable of simultaneously binding HER2 and a receptor expressed at the blood–brain barrier (BBB-R). This design leverages receptor-mediated transcytosis as a proposed strategy to enhance antibody transport across the BBB, addressing a long-standing limitation of HER2-targeted therapies in the treatment of central nervous system metastases. The patent covers molecular design, production methods, and potential therapeutic applications, illustrating a new generation of antibodies integrating HER2 targeting with spatially guided delivery.

Recent antibody innovations reflect a strategic shift from purely enhancing receptor blockade toward optimizing delivery, convenience, and tissue penetration. Subcutaneous fixed-dose formulations directly improve patient experience without altering biological efficacy, whereas BBB-penetrating trispecific antibodies address an unmet clinical need in CNS disease. These advances highlight that translational impact increasingly arises from engineering solutions to anatomical and logistical barriers rather than from further intensification of HER2 signaling inhibition.

Collectively, these patent disclosures illustrate the technological evolution of anti-HER2 monoclonal antibody–based strategies—from optimization of established therapeutic regimens, through formulation and delivery improvements, to advanced molecular engineering aimed at overcoming anatomical barriers. Importantly, these innovations represent development directions and enabling technologies rather than independent demonstrations of clinical efficacy. Detailed patent information is summarized in Table 4.

#### 3.2.4. Combination Therapy Strategies in HER2-Positive Breast Cancer

In recent years, the therapeutic development of HER2-positive breast cancer has increasingly focused on combination strategies explored at the translational and technological level, integrating HER2 blockade with modulation of key intracellular signaling pathways and immune responses. These approaches are proposed to address mechanisms of secondary resistance, including activation of the PI3K/AKT/mTOR axis, RAS/MAPK signaling, and tumor-driven immune evasion. Between 2015 and 2025, numerous patent filings illustrate that innovative activity in this field has concentrated on combinations linking anti-HER2 antibodies or antibody–drug conjugates (ADCs) with kinase inhibitors and immunomodulatory agents. The following examples are derived from patent disclosures and are discussed to illustrate emerging combination concepts rather than clinically validated therapeutic strategies.

Patent WO2025122745A1 [100] describes a proposed combination concept involving the PI3K inhibitor inavolisib (GDC-0077) with the fixed-dose subcutaneous formulation of pertuzumab and trastuzumab (PH FDC SC) administered on a 21-day schedule. This approach is designed to simultaneously target HER2-driven signaling and the PI3K/AKT/mTOR pathway and is intended for molecularly defined HER2-positive tumors harboring PIK3CA mutations, which are associated with reduced sensitivity to HER2 blockade. The patent exemplifies the development of genomically informed combination concepts aimed at pathway co-inhibition.

A similar development direction is reflected in US20230310455A1 [101], which outlines conceptual combinations of inavolisib with trastuzumab, pertuzumab, or their co-formulations. The disclosure emphasizes coordinated dosing strategies and alignment of administration schedules, highlighting PI3K inhibition as a proposed approach to explore restoration of sensitivity to anti-HER2 therapies.

Another prominent innovation trend involves the integration of HER2-targeted therapy with immunomodulation. WO2025164597A1 [95] describes a combination concept pairing anti-HER2 ADCs with immune checkpoint inhibitors or a STING agonist. This strategy is proposed to couple ADC-mediated cytotoxicity with immune activation within the tumor microenvironment. A related concept is presented in CN119654169A [102], which outlines a conceptual combination of an anti-HER2 ADC with a bispecific immune checkpoint inhibitor, aiming to explore synergistic engagement of tumor-cell targeting and immune modulation.

Among downstream signaling–oriented strategies, patents JP2025503511A [103] and KR20230042055A [104] describe proposed molecular co-targeting approaches. The former outlines a combination of an anti-HER2 ADC with a RAS G12C inhibitor, designed to simultaneously suppress mutant RAS signaling and deliver a cytotoxic payload. The latter focuses on combining anti-HER2 ADCs with inhibitors of HER receptor dimerization, particularly HER2/HER3 or HER2/EGFR complexes, which are implicated in secondary resistance mechanisms.

Finally, US12281153B2 [105] presents a targeted immunotherapy concept combining a trimeric 4-1BB (CD137) agonist with anti-HER2 antibodies, including trastuzumab, pertuzumab, or trastuzumab emtansine. This approach is designed to enhance antibody-mediated antitumor immune responses through T-cell co-stimulation, while aiming to mitigate systemic toxicity associated with conventional 4-1BB agonists.

Combination strategies integrating HER2 blockade with PI3K inhibition or immune modulation reflect the recognition that monotherapy is insufficient for durable disease control. While many proposed combinations remain preclinical or early clinical, they illustrate a rational convergence of targeted cytotoxicity and immune engagement. The primary translational challenge lies in identifying patients most likely to benefit while avoiding cumulative toxicity. Successful implementation of such strategies will depend on biomarker-guided selection and careful sequencing rather than universal application.

Collectively, these patent disclosures illustrate the direction of technological and translational innovation in HER2-positive breast cancer, moving from single-pathway targeting toward multi-axis combination concepts integrating receptor blockade, intracellular kinase inhibition, and immune modulation. Importantly, these strategies represent proposed development directions rather than established clinical standards. Detailed information on the referenced patent applications is summarized in Table 5.

### 3.3. Triple-Negative Breast Cancer (TNBC)

#### 3.3.1. Introduction and Biological Significance

TNBC is defined by the absence of ER and PR expression and the lack of HER2 amplification/overexpression; clinically, it accounts for approximately 10–15% of breast cancer diagnoses and is characterized by rapid growth kinetics, early relapse, and poorer prognosis compared with other subtypes [106]. Importantly, TNBC is not biologically uniform: transcriptomic profiling reveals stable molecular subtypes with distinct oncogenic drivers and therapeutic vulnerabilities. The most frequently cited classifications include TNBCtype-4 (BL1, BL2, M, LAR) [107] and the four Burstein subtypes (BLIS, BLIA, MES, LAR), where the immunologically “active” BLIA subtype is associated with improved outcomes, while the “immunosuppressive” BLIS subtype confers poorer prognosis [108]. Multi-omic analyses confirm that proliferative signaling (BL1/BL2), EMT program activation and mesenchymal signaling (M), and androgen receptor–driven transcriptional activity (LAR) form a functional landscape that shapes clonal evolution trajectories and resistance patterns in TNBC [109].

The TNBC genome is characterized by profound instability, with very high TP53 mutation rates (approximately 70–90%), frequent loss of PTEN and RB1, structural rearrangements, and copy-number alterations (e.g., 1q/8q gains, 5q/8p losses), as well as a lower prevalence of PIK3CA mutations compared with HR-positive breast cancer [110]. A substantial proportion of TNBC exhibits homologous recombination deficiency (HRD)—driven by BRCA1/2 mutations or a broader “BRCAness” genomic phenotype—which underlies sensitivity to PARP inhibitors and platinum agents and contributes to an enriched neoantigen landscape [111,112]. Concurrently, the TNBC immune microenvironment is often “hot”: high levels of tumor-infiltrating lymphocytes (TILs) correlate with improved outcomes and higher rates of pCR following neoadjuvant chemotherapy, while PD-L1 expression identifies subpopulations potentially sensitive to immunotherapy [113,114].

The biology of TNBC is also linked to a distinct propensity for central nervous system dissemination. In advanced disease, the risk of brain metastases is markedly elevated, reflecting both aggressive tumor kinetics and specific signaling programs (including PI3K/AKT, JAK/STAT, and EMT), while pharmacokinetic barriers such as the blood–brain barrier (BBB) remain major limitations to systemic efficacy [115]. Collectively, TNBC represents a cancer model defined by extensive heterogeneity, HRD dependence, and intrinsic immunogenicity, where clinical outcomes emerge from the complex interplay between genomic instability, inflammatory–immune status, and the evolutionary dynamics of the tumor.

Over the past decade, the therapeutic landscape of TNBC has evolved substantially, shifting from exclusive reliance on conventional chemotherapy toward strategies incorporating immunomodulation, DNA damage repair targeting, and antibody–drug conjugates (ADCs). The most dynamic areas of development include: (I) immunotherapy with immune checkpoint inhibitors (PD-1/PD-L1) and costimulatory agonists; (II) therapies targeting homologous recombination defects, including PARP inhibitor monotherapy as well as PARP inhibitors in combination with cytotoxic agents or antibody–drug conjugates (ADCs); and (III) ADCs directed against surface antigens characteristic of TNBC, such as TROP2, LIV-1, and Nectin-4. As in the HER2-positive subtype, many advances in TNBC are driven by technology development and reflected in patent activity, encompassing conjugation platforms, novel linkers and cytotoxic payloads, as well as combination concepts with immunotherapy or DNA repair–targeted agents. Accordingly, the following subsections summarize selected patent disclosures from 2015 to 2025 that illustrate emerging therapeutic concepts and development directions in TNBC rather than established clinical efficacy.

#### 3.3.2. ADCs in the Treatment of TNBC

In recent years, the development of ADCs in TNBC has increasingly moved beyond the classical “target + toxin” paradigm toward technologically complex and multi-layered design concepts. These approaches seek to integrate modulation of DNA damage response pathways, alternative antibody formats, enhanced internalization, and tailored pharmacokinetic properties to address the biological complexity of TNBC. Patent disclosures illustrate how such concepts are being explored at the translational level rather than providing independent evidence of clinical efficacy. Patent US10918734B2 [116] describes a proposed combination concept in which the TROP2-targeted ADC sacituzumab govitecan is combined with a Rad51 inhibitor. The rationale outlined in the patent is based on concurrent delivery of SN-38–mediated DNA damage and pharmacologic interference with homologous recombination repair mechanisms. This approach is designed to explore whether inhibition of Rad51-dependent repair pathways may sensitize TNBC cells to topoisomerase I–induced DNA damage, particularly in tumors that have regained HRR proficiency. The patent exemplifies a broader development strategy integrating ADCs with DNA repair modulation in TNBC. Parallel to these efforts, technological innovation has focused on the miniaturization and re-engineering of antibody carriers. Patent CN112321715B [117] describes a platform of TROP2-targeting VHH nanobodies characterized by high stability, efficient internalization, and favorable physicochemical properties. Their reduced molecular size is proposed to facilitate more homogeneous tumor penetration in densely structured TNBC lesions. These nanobodies constitute a flexible technological scaffold that can be adapted for ADC construction or other targeted therapeutic formats. A complementary design strategy is presented in patent EP4532020A1 [118], which focuses on payload selection and linker chemistry in anti–Nectin-4 ADCs. Rather than employing auristatin-based toxins, the patent proposes camptothecin derivatives (exatecan, SN-38, DXd) conjugated via protease-cleavable and self-immolative linkers. This configuration is designed to allow controlled intracellular payload release while limiting extracellular toxicity. The disclosed constructs represent a technological alternative to established MMAE-based ADC platforms, with potential relevance for Nectin-4–expressing TNBC. The most advanced molecular engineering approach is described in patent JP6869218B2 [119], which outlines humanized anti–LIV-1 antibodies and corresponding ADCs incorporating site-specific S239C conjugation. This strategy enables controlled drug-to-antibody ratios, improved conjugate homogeneity, and predictable pharmacokinetic behavior. The patent positions LIV-1 as a target with relatively uniform expression across multiple tumor types, including TNBC, and proposes that such properties may support consistent ADC delivery despite variable internalization dynamics. Collectively, patent filings from 2021 to 2025 illustrate that innovation in TNBC-directed ADCs is progressing along several complementary technological axes, including integration with DNA repair modulation, adoption of nanobody-based carriers, transition toward camptothecin-derived payloads, and precision site-specific conjugation. These approaches represent proposed development directions rather than validated therapeutic standards, highlighting the role of patent activity as a window into emerging ADC design strategies. Detailed patent information is summarized in Table 6.

#### 3.3.3. Immunotherapy in TNBC

The development of immunotherapy in TNBC has evolved considerably over the past decade, expanding beyond classical immune checkpoint inhibition toward technologically advanced platforms that include costimulatory agonists, immunomodulatory antibody conjugates, and miniaturized antibody or peptide-based formats. Patent disclosures from 2015 to 2025 illustrate this evolution at the conceptual and technological level, reflecting a shift from simple T-cell disinhibition toward increasingly precise engineering of the TNBC tumor microenvironment.

One major area of innovation continues to be the refinement of PD-1/PD-L1–directed strategies. Patent US10669338B2 [120] describes a new generation of PD-1–blocking antibodies defined by distinct CDR sequences, designed to optimize inhibition of PD-1/PD-L1 interactions. The patent further outlines their proposed integration into combination regimens with ADCs, type I interferons, or additional immune checkpoint inhibitors, reflecting broader development efforts aimed at combining checkpoint blockade with agents that modulate DNA damage or innate immune signaling. In parallel, patent ES3035911T3 [121] presents a conceptual antibody-independent approach based on small-molecule PD-1/PD-L1 inhibitors. These compounds are proposed to offer improved tissue penetration compared with large biologics, addressing pharmacokinetic limitations and enabling alternative combination strategies within the framework of checkpoint inhibition.

Beyond classical checkpoint blockade, increasing attention is being directed toward direct activation of innate immune pathways within the tumor microenvironment. Patent WO2023154318A1 [122] describes a conceptual immunoconjugate platform in which an anti-TROP2 antibody is linked to a small-molecule TLR7/8 agonist. Rather than delivering a cytotoxic payload, this “immunostimulatory ADC” is designed to target innate immune activation selectively to TROP2-expressing tumors. The disclosed strategy exemplifies a shift toward antibody-mediated delivery of immune stimulants as a means of locally modulating antigen presentation and inflammatory signaling within TNBC lesions.

Additional immunotherapeutic concepts focus on TNBC-associated surface antigens. Patent US11274160B2 [123] describes the unconjugated monoclonal antibody 14A5.2 directed against Nectin-4. The patent proposes that targeting Nectin-4 may interfere with tumor-cell adhesion and migratory processes, suggesting a potential role for anti–Nectin-4 antibodies beyond their use as ADC carriers. Such approaches highlight the exploration of antibody-based strategies aimed at modulating tumor biology independently of cytotoxic payload delivery.

A further development direction involves miniaturized costimulatory agonists. Patent US11613560B2 [124] introduces bicyclic OX40 agonist peptides designed to achieve robust T-cell co-stimulation through compact, chemically cyclized structures. Their reduced molecular size is proposed to facilitate tissue penetration and flexible molecular assembly, including multimeric formats or conjugation with additional functional moieties. These constructs illustrate an emerging class of non-antibody immune agonists explored as alternatives to conventional agonistic antibodies.

Collectively, these patent disclosures illustrate that immunotherapy development in TNBC is moving toward increasingly targeted, modular, and multi-functional concepts, encompassing checkpoint inhibition, innate immune activation, antigen-directed antibody strategies, and miniaturized costimulatory platforms. Importantly, these patents reflect technological exploration and development directions rather than established clinical efficacy. A summary of the referenced patent applications is provided in Table 7.

#### 3.3.4. Combination Therapies in TNBC

The development of combination therapies in TNBC is increasingly oriented toward integrated, multi-mechanistic strategies explored at the translational and technological level, aiming to combine targeted cytotoxicity, modulation of genomic instability, and immune microenvironment remodeling. Patent applications published between 2021 and 2025 illustrate this shift by proposing combination concepts designed to address resistance mechanisms that have limited the activity of monotherapies, including ADCs, DNA damage–response inhibitors, and immunotherapies.

Patent JP7608164B2 [125] describes a proposed combination platform in which exatecan-derived antibody–drug conjugates (DXd, topoisomerase I inhibitors) are combined with PARP inhibitors. The conceptual rationale is based on complementary induction of DNA damage, whereby ADC-delivered topoisomerase I inhibition is paired with pharmacologic interference with single-strand break repair. This strategy is designed to explore whether coordinated targeting of distinct DNA damage pathways may enhance genomic stress in tumor cells, including those without classical homologous recombination deficiency. The patent positions this approach as a platform-level concept applicable to multiple TNBC-relevant ADC targets, such as TROP2, LIV-1, and Nectin-4.

A complementary direction involves modulation of tumor immunometabolism. Patent TW202345845A [126] outlines a conceptual combination strategy pairing TROP2-targeted topoisomerase I–based ADCs, including sacituzumab govitecan, with inhibitors of the adenosine signaling axis, such as A2A/A2B receptor antagonists or CD39/CD73 inhibitors. The proposed approach seeks to integrate targeted DNA damage with reversal of adenosine-mediated immunosuppression within the tumor microenvironment, thereby exploring coordinated cytotoxic and immunomodulatory effects in TROP2-expressing TNBC.

Epigenetically informed combination strategies are presented in patent TWI816881B [127], which describes the use of BET bromodomain inhibitors in combination with PARP inhibitors, with optional inclusion of PD-1/PD-L1 blockade. The disclosed concept is based on epigenetic modulation of DNA damage–response gene expression, including pathways involved in homologous recombination and resistance to PARP inhibition. This approach is proposed to explore pharmacologic induction of HRD-like states in BRCA-wild-type TNBC, with the optional addition of immune checkpoint inhibition reflecting broader interest in integrating epigenetic and immunologic modulation.

In parallel, patent US11001628B2 [128] presents a dual-pathway immunotherapy concept combining PD-1 blockade with inhibition of the M-CSF signaling axis. This strategy is designed to concurrently relieve T-cell inhibition and reduce immunosuppressive macrophage populations within the tumor microenvironment. The patent outlines this approach as a means of addressing macrophage-driven resistance mechanisms that may limit the activity of immune checkpoint inhibitors in tumors with high myeloid infiltration, including TNBC.

Collectively, these patent disclosures illustrate that combination therapy development in TNBC is moving toward layered, multi-axis concepts integrating ADC-mediated cytotoxicity, DNA repair modulation, epigenetic sensitization, and immune microenvironment remodeling. Importantly, these patents represent proposed development directions and technological frameworks rather than validated clinical strategies. Detailed information on the selected patent applications is provided in Table 8.

The stage of translation and anticipated clinical impact were assigned based on the therapeutic modality, existing clinical validation of the platform, and the intended scope of each patent, as described in the original patent documents.

Importantly, several of the patented technologies discussed in this review are directly linked to therapeutic platforms that have already demonstrated clinical efficacy across different stages of clinical development. For instance, topoisomerase I inhibitor–based antibody–drug conjugates (ADCs) employing the exatecan/DXd payload platform—described in multiple patents from Daiichi Sankyo—have translated into clinically approved agents, most notably trastuzumab deruxtecan. This ADC has demonstrated robust efficacy in multiple Phase III clinical trials and continues to be evaluated in confirmatory Phase III and post-approval Phase IV studies across diverse HER2-positive and HER2-low breast cancer populations, including patients with brain metastases.

Similarly, patents describing TROP2-targeted ADC strategies align with the clinical development of sacituzumab govitecan, which has achieved regulatory approval following positive Phase III trials in metastatic triple-negative breast cancer and hormone receptor–positive disease. Importantly, several patents extend these validated ADC backbones into rational combination strategies, such as integration with DNA-damage–response inhibitors, reflecting an ongoing translational effort to enhance durability of response and overcome acquired resistance. Beyond approved ADCs, multiple patent families correspond to therapeutic concepts currently under active clinical investigation. These include combinations of anti-HER2 therapies with PI3K pathway inhibitors, particularly in the context of PIK3CA-mutant disease. Notably, PI3K-targeted agents such as inavolisib are being evaluated in late-stage Phase III clinical trials in combination regimens, supporting the clinical relevance of the resistance-oriented strategies described in these patents. In parallel, patents proposing the combination of ADCs with immune-modulating agents—including immune checkpoint inhibitors, co-stimulatory agonists, or innate immune activators—reflect a rapidly expanding area of early-phase clinical exploration. While many of these approaches remain in Phase I or Phase I/II trials, they exemplify the translational trajectory from mechanistic rationale to first-in-human evaluation. Finally, a subset of patents describes enabling or next-generation technologies, such as trispecific or blood–brain barrier–penetrating antibodies and advanced ADC manufacturing platforms, which currently reside at a preclinical or early Phase I stage but are poised to inform future clinical trial design. Together, these observations indicate that the patents analyzed reflect the full spectrum of translational oncology research—from clinically proven therapies, through late-stage clinical development, to early-stage innovations—underscoring the close relationship between patent activity and ongoing clinical trials.

### 3.4. Photodynamic Therapy (PDT): An Emerging Adjunctive and Translational Approach

PDT in breast cancer is based on three components: a photosensitizer that preferentially accumulates in the tumor, a light source of an appropriate wavelength, and molecular oxygen. At present, photodynamic therapy is not a standard option for systemic breast cancer treatment and is primarily applied as a local or adjunctive modality. Upon excitation, the photosensitizer generates reactive oxygen species that induce localized damage to membranes, proteins, and DNA, resulting in necrosis or apoptosis of tumor cells, shutdown of the tumor microvasculature, and strong inflammatory–immunological signaling [129]. Historically, PDT in breast cancer has been regarded as a niche modality—used primarily for palliation of cutaneous recurrences and chest-wall lesions after exhaustion of surgery, radiotherapy, and standard chemotherapy. A series of small clinical studies and case reports from the past decade confirm that sodium porfimer or 5-ALA can provide meaningful local responses and symptom relief (pain, exudation, bleeding) in patients with extensive, often irradiated chest-wall infiltrations, with an acceptable toxicity profile (treatment-related pain, transient phototoxic burns) [130]. However, its application is limited by the shallow depth of light penetration (typically 5–10 mm) and heterogeneous photosensitizer distribution, which restrict classical PDT to superficial lesions and adjunctive use rather than definitive treatment of primary breast tumors.

After 2015, the landscape of PDT research in breast cancer began to shift rapidly toward more “systemic” and technologically advanced strategies. Recent reviews highlight that PDT has evolved from a simple tissue-destructive technique to a modular theranostic platform in which photosensitizers are combined with targeting ligands, nanocarriers, or molecularly targeted drugs [131,132]. Nanocarriers (liposomes, polymers, gold nanorods, mesoporous silica) enhance the solubility of hydrophobic photosensitizers, prolong circulation time, and improve tumor accumulation via the EPR effect. Numerous preclinical breast cancer models—both ER-positive and triple-negative—demonstrate that nanoparticle-based delivery systems, such as curcumin or chlorin e6 complexes with metal or polymeric nanoparticles, significantly increase phototoxic efficacy and reduce systemic toxicity compared with free photosensitizers [133,134,135]. In TNBC, characterized by high vascular density and a tendency for cutaneous involvement, such photonanodrug systems appear particularly promising, enabling localized ablation with improved distribution within heterogeneous tumor tissue.

In parallel, the concept of photoimmunotherapy is emerging, combining antigen-specific recognition with spatially controlled light activation. In near-infrared photoimmunotherapy (NIR-PIT), an antibody against a tumor antigen (e.g., HER2) is conjugated to an NIR-absorbing dye (IR700); following intravenous administration and binding to tumor cells, NIR illumination induces rapid disruption of cell membranes exclusively in the irradiated area. In HER2-positive breast cancer models, trastuzumab–IR700 conjugates have been shown to produce rapid tumor reduction with striking “super-selectivity,” dependent on light dose and associated with minimal off-target toxicity [136]. These constructs functionally resemble ADCs, but instead of releasing a chemical cytotoxic payload, they convert light energy into a cytolytic effect—offering a potential strategy for lesions resistant to trastuzumab or T-DM1 while maintaining high antigen specificity. Similar approaches are now being adapted to targets relevant to breast cancer (EGFR, EpCAM), and their translation into early-phase clinical trials represents one of the most rapidly developing areas of next-generation PDT.

A key aspect distinguishing modern PDT from classical photochemotherapy is the expanding understanding of its immunological consequences. ROS-induced damage triggers immunogenic cell death (ICD), characterized by exposure of DAMPs (calreticulin, HMGB1, ATP), activation of dendritic cells, and generation of T-cell responses directed against tumor antigens. Recent work in metastatic TNBC models shows that the use of an “immunomodulatory” photosensitizer capable of inducing strong ICD can markedly potentiate PD-1/PD-L1 blockade, leading to regression not only of the irradiated tumor but also distant lesions (abscopal effect), along with durable immunological memory [137]. Thus, combining PDT with checkpoint inhibitors, cancer vaccines, or even immunostimulatory ADCs (e.g., anti-TROP2–TLR7/8) represents a logical step toward converting breast cancer—including TNBC—from an immunologically “cold” to a “hot,” therapy-responsive tumor phenotype.

Despite these advancements, the routine clinical use of PDT in breast cancer remains limited. Technical barriers include restricted light penetration in breast tissue (especially for photosensitizers absorbing at 630–660 nm), challenges in delivering a homogeneous light dose across large or irregular lesions, and the need for precise positioning of optical fibers for percutaneous or interstitial techniques. From a biological perspective, major challenges include heterogeneous photosensitizer distribution within tumors, the impact of hypoxia, and variability in antigen expression in the case of photoimmunotherapy. Conversely, the development of second- and third-generation photosensitizers absorbing in the deep NIR range, nanoparticle-based constructs, integration with fluorescence/photoacoustic imaging, and improved understanding of PDT–immune system interactions are transforming light-based therapy from a niche palliative modality into a credible candidate for incorporation into complex breast cancer treatment algorithms—both HER2-positive and triple-negative—as a component of local ablation, an “in situ vaccine,” and an amplifier of immunotherapy and molecularly targeted agents [129,132,137].

## 4. Discussion

The past decade has brought an unprecedented number of therapeutic innovations in breast cancer, leading to a profound transformation in both the biological understanding of the disease and clinical practice. The review presented in this work highlights three dominant processes driving this progress: (1) the technological evolution of targeted therapies and ADCs; (2) the integration of endocrine, immunologic, DDR-based, and signaling-pathway therapies; and (3) the expansion of the therapeutic landscape to include previously niche and investigational modalities such as PDT. Each breast cancer subtype—HR+/HER2−, HER2+, and TNBC—benefits from these innovations in distinct ways, illustrating the complexity and direction of change within breast oncology.

In HR+/HER2− disease, the past decade has confirmed the central role of targeted therapies as the backbone of treatment. CDK4/6 inhibitors have altered the natural history of first-line HR+/HER2− metastatic disease, with overall survival benefits now confirmed across additional populations, including those with aggressive clinical behavior. Simultaneously, this review shows that therapeutic development has not been linear: the failure of palbociclib in the adjuvant setting exposed the importance of MRD dynamics and proliferation variability, underscoring that even in a seemingly well-characterized subtype, clonal evolution remains a key determinant of outcomes. The growing relevance of PI3K/AKT/mTOR–directed therapies—particularly selective PI3Kα inhibitors and AKT inhibitors—emphasizes that overcoming endocrine resistance requires targeting broader signaling hubs than previously appreciated. Integration of oral SERDs, especially those guided by ESR1 mutations in ctDNA, represents a shift from a model of treating radiologic progression toward biomarker-driven adaptive therapy.

HER2-positive breast cancer has also undergone a major transformation. HER2 blockade is no longer defined by two antibodies and a single ADC—it now represents one of the most technologically advanced therapeutic domains in breast oncology. The patent landscape reviewed here highlights the increasing importance of molecular engineering: from subcutaneous trastuzumab/pertuzumab formulations, to trispecific antibodies actively transported across the BBB, to novel immune-modulatory strategies using 4-1BB agonists. The introduction of second-generation ADCs (DXd-based), along with emerging ADC approaches for patients with niche disease sites (e.g., CNS metastases), underscores that the future of this subtype lies not only in deeper HER2 inhibition but also in precise drug delivery to microenvironments with limited pharmacologic accessibility. This is particularly critical in the context of brain metastases—an area historically underserved by existing therapies.

TNBC, as the most biologically challenging subtype, reflects the full spectrum of change in cancer therapeutics. This review demonstrates that progress in TNBC arises not from a single breakthrough but from the convergence of multiple mechanisms: synthetic lethality (PARP inhibition, HRD), targeted cytotoxicity (ADC targeting TROP2, LIV-1, Nectin-4), epigenetic modulation (BET inhibition), next-generation immunotherapies (novel anti-PD-1 antibodies, small-molecule PD-1/PD-L1 inhibitors, OX40 agonists), and immunometabolic therapies (CD73/CD39/A2A blockade). Patent analyses confirm that innovation in TNBC is inherently multilayered—the most effective strategies do not strengthen a single pathway, but instead simultaneously target DDR, immunosuppression, and proliferation. This aligns with clinical observations, in which immunotherapy monotherapy has limited activity, whereas systemic reversal of immunosuppression—e.g., anti-PD-1 combined with ADCs or PARP inhibitors—produces markedly superior results. TNBC has become the domain where molecular, chemical, and immunologic technologies intersect most intensively, and the “innovation race” is now driven largely by patent development and drug-engineering advances.

Perhaps one of the most unexpected scientific developments of the past decade is the renewed research interest in PDT, which has evolved from a predominantly palliative technique into an advanced, yet still investigational, theranostic platform. In the context of breast cancer, PDT has regained translational relevance through the introduction of nanocarriers, dual diagnostic–therapeutic designs, near-infrared photoimmunotherapy (NIR-PIT), and photosensitizers capable of inducing immunogenic cell death. Particularly noteworthy are the interactions between PDT and immunotherapy in TNBC: induction of immunogenic cell death, activation of dendritic cells, and the potential to elicit abscopal effects position PDT not merely as a method of local ablation but as a candidate for an “in situ vaccine” strategy under preclinical and early translational investigation, capable of enhancing PD-1/PD-L1 inhibitors or immunostimulatory ADCs. PDT additionally illustrates how profoundly drug-delivery technologies—including nanoparticles, NIR dyes, and targeting systems—can influence therapeutic efficacy, a recurring theme throughout this review.

Despite these advances, important challenges persist across the breast cancer treatment continuum. In HR+/HER2− disease, an optimal sequencing algorithm following CDK4/6 inhibition remains undefined, particularly given the expanding number of potential PI3K/AKT/mTOR and SERD-based combinations. In HER2-positive disease, there is an urgent need to develop strategies specifically directed at central nervous system involvement, employing molecules capable of effectively crossing the blood–brain barrier. In TNBC, a major unresolved challenge remains the identification of robust biomarkers to guide patient selection for increasingly complex combination regimens. PDT, meanwhile, requires further standardization, miniaturization of delivery technologies, and clarification of its impact on long-term disease control. Additionally, many of the most technologically advanced approaches discussed in this review currently exist only at the patent or early developmental stage, underscoring that clinical translation remains a critical next step.

Taken together, this review illustrates that the past decade is redefining breast cancer therapy by shifting away from monotherapies toward complex, mechanistically synergistic strategies. The unifying thread across major therapeutic innovations—HR+/HER2−, HER2+, TNBC, as well as emerging adjunctive approaches such as PDT—is the drive to integrate targeted therapy, immunotherapy, and advanced drug-delivery technologies. This logic is likely going to continue shaping the evolution of breast cancer treatment in the coming years.

## 5. Conclusions and Future Directions

The past decade has brought transformative advances in breast cancer treatment, driven by the parallel evolution of molecular biology, drug engineering, and therapeutic delivery technologies. In HR+/HER2− disease, CDK4/6 inhibitors, modulators of the PI3K/AKT/mTOR pathway, and oral selective estrogen receptor degraders have become fundamental components of therapy, enabling increasingly effective strategies to overcome endocrine resistance and to adapt treatment dynamically to real-time molecular changes detected through liquid biopsy. In HER2-positive breast cancer, innovations in antibody and antibody–drug conjugate design—including next-generation constructs and antibodies capable of crossing the blood–brain barrier—have expanded therapeutic benefit to patient populations that were historically difficult to treat. In TNBC, the most biologically aggressive subtype, the field has undergone particularly rapid development, with advanced ADCs, next-generation immunotherapies, DDR inhibitors, and epigenetic agents forming multimodal platforms capable of simultaneously modulating immunity, DNA repair, and tumor-cell proliferation.

Concurrently, photodynamic therapy has evolved into a modern, yet still investigational, phototheranostic modality that is primarily applicable in localized or adjunctive settings. By incorporating nanocarriers, photoimmunotherapy, and photosensitizers capable of inducing immunogenic cell death, PDT is generating new opportunities for synergy with systemic immunotherapy at the preclinical and translational levels. Unlike antibody–drug conjugates and immunotherapy, PDT currently remains outside standard systemic treatment algorithms in breast cancer and should therefore be regarded as an adjunctive or exploratory strategy rather than a replacement for established systemic therapies. Collectively, the available evidence indicates that the future of breast cancer treatment will rely on the integration of multiple complementary approaches—targeted, immunologic, epigenetic, and localized—and on increasingly precise use of dynamic biomarkers such as ctDNA, alongside delivery technologies capable of reaching pharmacologically inaccessible tumor niches. The progress of recent years demonstrates that breast cancer is becoming a disease increasingly susceptible to complex, multilayered therapeutic interventions, and that further improvements in clinical outcomes will arise from the strategic integration of molecular, immunologic, and technological innovations, progressively shifting treatment from sequential monotherapy to personalized combinations tailored to dominant mechanisms of resistance. Over the past decade, breast cancer research has transitioned from pathway-centric drug development toward integrative, biomarker-driven therapeutic strategies. The most successful innovations—CDK4/6 inhibitors, selective PI3Kα inhibitors, next-generation ADCs, and oral SERDs—share a common feature: direct alignment between molecular mechanism and patient selection. Conversely, approaches lacking this alignment frequently failed despite strong preclinical rationale. Looking forward, translational progress will likely depend less on identifying new targets and more on optimizing therapeutic sequencing, overcoming resistance through anticipatory intervention, and integrating systemic and localized immune-modulating approaches. Patent activity increasingly reflects this shift, emphasizing delivery technologies, combination regimens, and adaptive treatment models. Together, these trends define a new phase of precision oncology in breast cancer, characterized not by single-agent breakthroughs but by intelligently layered therapeutic strategies.

## Figures and Tables

**Table 1:** Completed Clinical Trials of Cyclin-Dependent Kinase 4/6 (CDK4/6) Inhibitors in Advanced Hormone Receptor–Positive/Human Epidermal Growth Factor Receptor 2–Negative (HR+/HER2−) Breast Cancer.

Trial (NCT Number)	Year of Initiation/Publication	Phase	Population	Treatment Regimen (Intervention/Control)	Key Outcomes (PFS/OS)	Results	Reference
MONALEESA-2 (NCT01958021)	2013/2017	III	668 postmenopausal women with HR+/HER2− advanced breast cancer (ABC)	Ribociclib + letrozole vs. placebo + letrozole	PFS 25.3 vs. 16.0 months (HR 0.56); ORR 52.7% vs. 37.1%	Ribociclib + letrozole confirmed significant improvement in median PFS (25.3 vs. 16.0 months) and higher objective response, and the PFS benefit was seen regardless of molecular mutations; trend indicates improved survival in the ribociclib arm	[41,42]
MONALEESA-7 (NCT02278120)	2014/2018	III	672 premenopausal women with HR+/HER2− advanced breast cancer (ABC)	Ribociclib + ET + goserelin vs. placebo + ET	PFS 23.8 vs. 13.0 months; OS 58.7 vs. 48.0 months (HR 0.76)	In women with HR+/HER2− advanced breast cancer, ribociclib + ET demonstrated a significant prolongation of PFS and a median OS of 58.7 vs. 48.0 months, confirming the therapeutic benefit in this population	[43,44]
MONALEESA-3 (NCT02422615)	2015/2018	III	Postmenopausal women and men with advanced HR+/HER2− breast cancer after ≤1 line of endocrine therapy	Ribociclib + fulvestrant vs. placebo + fulvestrant	PFS 20.5 vs. 12.8 months (HR 0.59); OS 67.6 vs. 51.8 months (HR 0.67)	Ribociclib + fulvestrant prolonged median PFS (20.5 vs. 12.8 months) and median OS (67.6 vs. 51.8 months) in first-line treatment, confirming a durable treatment benefit	[45,46]
CompLEEment-1 (NCT02941926)	2016/2022	IIIb	>3200 patients with HR+/HER2− advanced breast cancer (ABC), mixed menopausal status, ECOG ≤ 2	Ribociclib + letrozole ± goserelin/leuprolide	TTP 27.1 months; CBR 78%; QoL maintained	Ribociclib + letrozole ± goserelin/leuprolide in the real-world population confirmed a long time to progression (TTP ≈ 27.1 months) and high clinical benefit (CBR ~78%), with a consistent safety profile and maintained quality of life	[47]
RIGHT Choice (NCT03839823)	2019/2023	II	222 pre- and perimenopausal women with aggressive HR+/HER2− advanced breast cancer (ABC)	Ribociclib + ET + goserelin vs. CT (docetaxel + capecitabine/paclitaxel + gemcitabine/capecitabine + vinorelbine)	PFS 21.8 vs. 12.8 months (HR 0.61); ORR 66.1% vs. 61.8%	Ribociclib + ET significantly prolonged median PFS versus chemotherapy combination (21.8 vs. 12.8 months; HR0.61), with similar response rates and better tolerability, demonstrating clinical efficacy in an aggressive population.	[48]
PEARL (NCT02028507)	2014/2022	III	601 postmenopausal women with HR+/HER2− metastatic breast cancer (MBC), AI-resistant	Palbociclib + ET vs. capecitabine	PFS 7.5 vs. 10.0 months (NS); OS 34.0 vs. 29.2 months	In the aromatase inhibitor-resistant population, palbociclib + ET did not improve PFS or OS compared with capecitabine; data suggest similar survival outcomes, although quality of life improvement was more favorable in the palbociclib arm	[49]
AMALEE (NCT03822468)	2019/2024	II	376 women with HR+/HER2− breast cancer (pre- and postmenopausal, 1L)	Ribociclib 400 mg vs. 600 mg + NSAI ± goserelin	PFS 26.9 vs. 25.1 months; DOR 26.5 vs. 28.8 months	Dose comparison of ribociclib 400 mg vs. 600 mg with NSAI showed comparable PFS and DOR between doses, with lower rates of neutropenia and QTc prolongation in the lower dose group, supporting the use of 400 mg in clinical practice.	[50]

Abbreviations: CDK4/6—cyclin-dependent kinase 4/6; HR+—hormone receptor–positive; HER2−—human epidermal growth factor receptor 2–negative; ABC—advanced breast cancer; MBC—metastatic breast cancer; ET—endocrine therapy; AI—aromatase inhibitor; PFS—progression-free survival; OS—overall survival; ORR—objective response rate; TTP—time to progression; CBR—clinical benefit rate; QoL—quality of life; CT—chemotherapy; NS—not significant; 1L—first-line treatment.

**Table 2:** Completed clinical trials of phosphatidylinositol 3-kinase/protein kinase B/mechanistic target of rapamycin (PI3K/AKT/mTOR) inhibitors in advanced hormone receptor–positive/human epidermal growth factor receptor 2–negative (HR+/HER2−) breast cancer.

Trial (NCT Number)	Year of Initiation/Publication	Phase	Population	Treatment Regimen (Intervention/Control)	Key Outcomes (PFS/OS)	Results	Reference
FERGI (NCT01437566)	2011/2016	II	Postmenopausal ER+/HER2− ABC/MBC resistant to AI; Cohort 1: PIK3CA–unselected; Cohort 2: PIK3CA-mutation	Pictilisib 340 mg (Cohort 1) or 260 mg (Cohort 2) + fulvestrant vs. placebo + fulvestrant.	PFS Cohort 1: 6.6 vs. 5.1 months (HR 0.74; *p* = 0.096); Cohort 2: 5.4 vs. 10.0 months (HR 1.07; *p* = 0.84); OS: no improvement.	Addition of pictilisib to fulvestrant did not significantly improve progression-free survival compared with placebo plus fulvestrant in either the overall or PIK3CA-mutated cohort, and was limited by toxicity without demonstrating a meaningful efficacy benefit.	[64]
SOLAR-1 (NCT02437318)	2015/2019	III	572 patients (postmenopausal women/men) with HR+/HER2− ABC after prior ET; 341 with PIK3CA mutation.	Alpelisib 300 mg QD + fulvestrant 500 mg vs. placebo + fulvestrant.	PIK3CA-mut: PFS 11.0 vs. 5.7 months (HR 0.65; 95% CI 0.50–0.85; *p* < 0.001); PIK3CA-wt: HR 0.85 (95% CI 0.58–1.25); OS: not reached (2019 analysis).	In patients with PIK3CA-mutated HR+/HER2− advanced breast cancer, alpelisib + fulvestrant nearly doubled median PFS (11.0 vs. 5.7 months; HR 0.65) compared with placebo plus fulvestrant, with a trend toward numerically longer overall survival; improvements in ORR and clinical benefit rate were also observed.	[18]
BYLieve (NCT03056755)	2017/2024	II	379 patients with HR+/HER2− ABC/MBC and PIK3CA mutation after progression on CDK4/6 + AI (Cohort A), CDK4/6 + FUL (Cohort B), or prior ET/CT without CDK4/6 exposure (Cohort C).	Alpelisib 300 mg QD + FUL (Cohorts A, C) or LET (Cohort B); no control arm.	PFS: A 7.3 months (95% CI 5.6–8.3); B 5.6 months (95% CI 3.7–7.3); C 12.0 months (95% CI 9.2–16.8); ≥50% progression-free at 6 months (Cohort A); OS: medians not reached (2024 cut-off).	Alpelisib plus endocrine therapy demonstrated clinically meaningful activity in PIK3CA-mutated HR+/HER2− advanced breast cancer after progression on prior therapies, with a substantial proportion of patients alive without progression at 6 months and manageable toxicity in the post-CDK4/6 inhibitor setting.	[65]
XENERA-1 (NCT03659136)	2018/2022	II	HR+/HER2− MBC with non-visceral disease; prior HT ± CDK4/6 inhibitor.	Xentuzumab 1000 mg IV qw + everolimus 10 mg + exemestane 25 mg vs. placebo + everolimus + exemestane.	PFS (independent assessment): 12.7 vs. 11.0 months (HR 1.19; *p* = 0.6534); PFS (investigator-assessed): 7.4 vs. 9.2 months (HR 1.23; *p* = 0.48); OS: no benefit.	The addition of xentuzumab to everolimus and exemestane did not confer a PFS advantage over everolimus and exemestane alone; median PFS was similar between arms per independent and investigator assessment, and no clear benefit was observed with the investigational agent	[66]

Abbreviations: ABC—advanced breast cancer; MBC—metastatic breast cancer; AI—aromatase inhibitor; ET—endocrine therapy; FUL—fulvestrant; LET—letrozole; PFS—progression-free survival; OS—overall survival; QD—once daily; IV—intravenous; qw—once weekly; HR—hazard ratio; CI—confidence interval; HT—hormone therapy; CT—chemotherapy; CDK4/6—cyclin-dependent kinase 4/6; PIK3CA-mut—PIK3CA mutation; PIK3CA-wt—PIK3CA wild type; PFS—Progression-Free Survival; OS—Overall Survival.

**Table 3:** Overview of selected patent applications related to human epidermal growth factor receptor 2–targeted antibody–drug conjugates (HER2-targeted ADCs) in the treatment of breast cancer.

Patent Number	Year of Publication	Assignee (Company)	Technology/Application	Proposed Technological Innovation	Stage of Translation	Anticipated Clinical Impact	Status	Reference
AU2025201016A1	2025	Daiichi Sankyo Co., Ltd. (Tokyo, Japan)	Anti-HER2 ADC concept	Peptide linker “GGFG” + DX payload conjugated to an anti-HER2 antibody; proposed as a next-generation ADC concept aimed at addressing heterogeneous HER2 expression and resistance mechanisms	Clinically validated ADC platform (derivative innovation)	Moderate–High (HER2-low, resistance)	Pending	[94]
WO2025164597A1	2025	Daiichi Sankyo Co., Ltd. (Tokyo, Japan)	ADC-based combination concept)	Conceptual combination of anti-HER2 ADCs with immune checkpoint inhibitors (PD-1/PD-L1, CTLA-4), proposed to explore strategies for modulating antitumor immunity and addressing resistance pathways in HER2-positive disease	Early clinical/preclinical	Potentially transformative (if tolerable)	Pending	[95]
JP7674542B2	2025 (grant: 9 May 2025)	Daiichi Sankyo Co., Ltd. (Tokyo, Japan)	ADC–manufacturing and purification technology (exatecan/T-DXd platform)	Optimized ADC purification and manufacturing process designed to reduce protein aggregation and impurities, improve conjugate stability, and support scalable production of next-generation ADC platforms	Clinically validated manufacturing platform	Incremental (scalability, quality)	Active	[96]

Abbreviations: ADC—antibody–drug conjugate; HER2—human epidermal growth factor receptor 2; DX—DXd, topoisomerase I inhibitor payload (used in trastuzumab deruxtecan, T-DXd); T-DXd—trastuzumab deruxtecan; GGFG—peptide linker sequence (glycine–glycine–phenylalanine–glycine).

**Table 4:** Overview of selected patent applications related to human epidermal growth factor receptor 2–targeted (HER2-targeted) monoclonal antibodies in breast cancer therapy.

Patent Number	Year of Publication	Assignee (Company)	Technology/Application	Proposed Technological Innovation	Stage of Translation	Anticipated Clinical Impact	Status	Reference
US20250018032A1	2025	Genentech Inc.; (South San Francisco, CA, USA) Hoffmann-La Roche Inc. (Little Falls, NJ, USA)	Anti-HER2 monoclonal antibody–based adjuvant treatment strategy (pertuzumab + trastuzumab)	Patent-protected therapeutic application defining dosing and administration strategies for dual HER2 blockade in the adjuvant setting, supporting intensified pathway inhibition in high-risk early HER2-positive breast cancer	Clinically validated standard of care	High (DFS, OS benefit)	Pending	[97]
US20240269064A1	2024	Genentech Inc.; (South San Francisco, CA, USA) Hoffmann-La Roche Inc. (Little Falls, NJ, USA)	Monoclonal antibodies–subcutaneous formulations of pertuzumab and trastuzumab	Fixed-dose subcutaneous co-formulations of pertuzumab and trastuzumab designed to streamline administration and improve treatment convenience while preserving antibody integrity	Clinically implemented formulation	Moderate (patient convenience)	Pending	[98]
US20230212312A1 → converted to US12331134B2 (granted 2025)	2023 (A1), 2025 (B2–granted)	Hoffmann-La Roche Inc. (Little Falls, NJ, USA)	Trispecific antibody targeting HER2 and a blood–brain barrier receptor	Engineered trispecific antibody construct incorporating a blood–brain barrier receptor–binding domain, proposed to facilitate receptor-mediated transcytosis and improve central nervous system exposure of HER2-targeted antibodies.	Early clinical/advanced preclinical	Potentially transformative (brain metastases)	Granted (US12331134B2)	[99]

Abbreviations: HER2—human epidermal growth factor receptor 2; A1—patent application; B2—granted patent publication.

**Table 5:** Summary of selected patent applications concerning human epidermal growth factor receptor 2–targeted (HER2-targeted) combination therapies in the treatment of breast cancer.

Patent Number	Year of Publication	Assignee (Company)	Technology/Application	Proposed Technological Innovation	Stage of Translation	Anticipated Clinical Impact	Status	Reference
WO2025122745A1	2025	Genentech Inc.; (South San Francisco, CA, USA) Hoffmann-La (Roche Inc. (Little Falls, NJ, USA)	Combination of anti-HER2 monoclonal antibodies (PH FDC SC: pertuzumab + trastuzumab) with a PI3K inhibitor (inavolisib)	Fixed-dose subcutaneous pertuzumab/trastuzumab combined with an oral PI3K inhibitor (inavolisib), proposed to explore pathway co-inhibition in PIK3CA-mutant HER2-positive breast cancer	Early clinical	Moderate–High (resistance reversal)	Pending	[100]
US20230310455A1	2023	Genentech Inc.; (South San Francisco, CA, USA) Hoffmann-La (Roche Inc. (Little Falls, NJ, USA)	Combination of targeted therapies–PI3K inhibitor (inavolisib) plus anti-HER2 antibodies	Conceptual combination of inavolisib with trastuzumab and/or pertuzumab, designed to investigate coordinated dosing strategies and potential restoration of sensitivity to HER2-targeted therapy	Early clinical/translational	Moderate	Pending	[101]
WO2025164597A1	2025	Daiichi Sankyo Co., Ltd. (Tokyo, Japan)	Combination strategy (anti-HER2 ADC + immunotherapy/STING agonist)	Concurrent administration of an anti-HER2 ADC with immune checkpoint inhibitors or a STING agonist, proposed to explore combined cytotoxic and immune-activating mechanisms	Preclinical/early translational	Potentially transformative	Pending	[95]
CN119654169A	2025	AstraZeneca UK Ltd.; (Cambridge, UK) Daiichi Sankyo Co., Ltd. (Tokyo, Japan)	Combination of anti-HER2 ADC with a bispecific immune-checkpoint inhibitor	Conceptual combination of a HER2-targeted ADC with a bispecific immune checkpoint inhibitor to explore synergistic tumor targeting and immune modulation	Preclinical	Uncertain–Moderate	Pending	[102]
JP2025503511A	2025	Daiichi Sankyo Co., Ltd. (Tokyo, Japan)	Combination of anti-HER2 ADC with a RAS-G12C inhibitor	Proposed co-targeting strategy combining an anti-HER2 ADC with a RAS G12C inhibitor to address downstream signaling resistance	Preclinical	Uncertain (niche population)	Pending	[103]
KR20230042055A	2023	Daiichi Sankyo Co., Ltd. (Tokyo, Japan)	Combination of an anti-HER2 ADC with a HER-dimerization inhibitor	Conceptual combination of an anti-HER2 ADC with a HER dimerization inhibitor aimed at disrupting HER2−HER3/EGFR–mediated resistance mechanisms	Preclinical	Moderate	Pending	[104]
US12281153B2	2025	Hoffmann-La Roche Inc. (Little Falls, NJ, USA)	Combination of a 4-1BB (CD137) agonist with anti-HER2 antibodies (trastuzumab, pertuzumab, T-DM1)	Combination of a trimeric 4-1BB (CD137) agonist with anti-HER2 antibodies, designed to enhance antibody-mediated immune activation while limiting systemic toxicity	Early clinical/translational	Potentially high	Active	[105]

Abbreviations: HER2—human epidermal growth factor receptor 2; ADC—antibody–drug conjugate; PH FDC SC—pertuzumab + trastuzumab fixed-dose combination subcutaneous formulation; PI3K—phosphatidylinositol 3-kinase; PIK3CA-mut—PIK3CA mutation; STING—stimulator of interferon genes; RAS-G12C—specific activating mutation of RAS protein (glycine 12 → cysteine); EGFR—epidermal growth factor receptor; 4-1BB (CD137)—co-stimulatory receptor on T lymphocytes; T-DM1—trastuzumab emtansine.

**Table 6:** Review of key patent applications concerning next-generation ADCs targeting TNBC.

Patent Number	Year of Publication	Assignee (Company)	Technology/Application	Proposed Technological Innovation	Stage of Translation	Anticipated Clinical Impact	Status	Reference
US10918734B2	2021	Immunomedics Inc. (Morris Plains, NJ, USA)	ADC (sacituzumab govitecan, TROP2-targeted) + Rad51 inhibitor (HRR/DDR combination)	Conceptual combination of sacituzumab govitecan with a Rad51 inhibitor, proposed to explore sensitization of TNBC cells to topoisomerase I–induced DNA damage through interference with homologous recombination repair pathways	Clinically validated ADC + translational combination	High (resistance mitigation, durability)	Active	[116]
CN112321715B	2022	Boaoxin Biotechnology Nanjing Co., Ltd. (Nanjing, Jiangsu, China)	Novel antibodies–TROP2-targeting nanobodies (VHH); platform suitable for ADC development	Development of high-affinity, strongly internalizing anti-TROP2 nanobodies (VHH) designed as a lightweight carrier platform for constructing deeply penetrating ADCs or other targeted therapeutic modalities	Preclinical/platform technology	Moderate (enabling future ADCs)	Active	[117]
EP4532020A1	2025	Innate Pharma SA (Marseille, France)	Anti-Nectin-4 ADCs with camptothecin-based payloads (exatecan, Dxd, SN-38) and protease-cleavable linkers	Humanized anti–Nectin-4 antibodies conjugated to camptothecin derivatives (exatecan, SN-38, DXd) via protease-cleavable linkers, proposed to enable controlled intracellular payload release and alternative ADC design relative to auristatin-based constructs	Early translational/preclinical ADC platform	Moderate–High (post-EV alternatives)	Pending	[118]
JP6869218B2	2021	Seagen Inc. (Bothell, WA, USA)	Humanized anti–LIV-1 antibodies and corresponding LIV-1–vcMMAE/LIV-1–mcMMAF ADCs (site-specific S239C conjugation)	Humanized anti–LIV-1 antibodies incorporating S239C site-specific conjugation to generate homogeneous ADCs with controlled drug-to-antibody ratios and predictable pharmacokinetic properties	Clinically validated ADC platform	Moderate (target diversification in TNBC)	Active	[119]

Abbreviations: ADC—antibody–drug conjugate; TNBC—triple-negative breast cancer; TROP2—trophoblast cell-surface antigen 2; HRR—homologous recombination repair; DDR—DNA damage response; SN-38—active metabolite of irinotecan; VHH—single-domain nanobody (variable heavy chain); vcMMAE/mcMMAF—valine-citrulline– or maleimidocaproyl-linked auristatin payloads; LIV-1—solute carrier family 39 member 6, a zinc transporter overexpressed in TNBC.

**Table 7:** Review of selected patent applications concerning immunotherapy approaches in TNBC.

Patent Number	Year of Publication	Assignee (Company)	Technology/Application	Proposed Technological Innovation	Stage of Translation	Anticipated Clinical Impact	Status	Reference
US10669338B2	2020	Immunomedics Inc. (Morris Plains, NJ, USA)	Anti-PD-1 antibodies; checkpoint inhibitors; potential combinations with ADCs/interferons/ICIs	Novel humanized PD-1 antibodies with defined CDR sequences, designed to optimize PD-1/PD-L1 blockade and proposed for integration into combination regimens with ADCs, interferons, or additional immune checkpoint inhibitors.	Clinically validated immunotherapy platform	High (combination-enabling backbone)	Active	[120]
ES3035911T3	2025	Gilead Sciences Inc. (Foster City, CA, USA)	Small-molecule inhibitors of PD-1/PD-L1	Development of small-molecule inhibitors targeting the PD-1/PD-L1 interaction, proposed as antibody-independent alternatives or complements enabling new checkpoint-based combination strategies.	Early translational/advanced preclinical	Moderate–High (access, combinations)	Active	[121]
WO2023154318A1	2023	Bolt Biotherapeutics Inc. (Redwood City, CA, USA)	Anti-TROP2 immunoconjugate with a TLR7/8 agonist (2-aminobenzazepine)	Targeted delivery of a TLR7/8 agonist to TROP2-positive tumors via an anti-TROP2 antibody, representing a conceptual “immunostimulatory ADC” platform designed to locally activate innate immune pathways.	Preclinical/early translational	Potentially transformative (if efficacy confirmed)	Ceased (PCT application)	[122]
US11274160B2	2022	INSERM, CNRS, Aix-Marseille Université, Institut Paoli-Calmettes (Marseille, France)	Unconjugated anti-Nectin-4 monoclonal antibody (14A5.2 mAb)	Unconjugated anti–Nectin-4 monoclonal antibody proposed to modulate tumor-cell adhesion and migratory behavior, explored as an antibody-based strategy independent of cytotoxic payload delivery.	Early clinical/translational	Moderate (metastasis suppression)	Active	[123]
US11613560B2	2023	BicycleTx Ltd. (Cambridge, UK)	Bicyclic OX40 agonist peptide (monomers, multimers, conjugates)	Bicyclic OX40 agonist peptides designed as compact, chemically cyclized costimulatory platforms enabling multivalent assembly or conjugation for targeted T-cell activation.	Early clinical/translational	Moderate–High (immune activation)	Active	[124]

Abbreviations: TNBC—triple-negative breast cancer; PD-1—programmed death-1; PD-L1—programmed death-ligand 1; ADC—antibody–drug conjugate; ICI—immune checkpoint inhibitor; TLR7/8—toll-like receptor 7/8; mAb—monoclonal antibody; OX40—co-stimulatory receptor on T lymphocytes.

**Table 8:** Review of selected patent applications concerning combination therapies in TNBC.

Patent Number	Year of Publication	Assignee (Company)	Technology/Application	Proposed Technological Innovation	Stage of Translation	Anticipated Clinical Impact	Status	Reference
JP7608164B2	2025	Daiichi Sankyo Co., Ltd. (Tokyo, Japan)	Exatecan-derived ADC (DXd platform) + PARP inhibitor (DDR combination therapy)	Conceptual combination of exatecan-derived ADCs with PARP inhibitors, designed to explore coordinated induction of DNA damage and interference with repair pathways across multiple TNBC-relevant ADC targets.	Early clinical/advanced translational	High (DDR-sensitized tumors)	Active	[125]
TW202345845A	2023	Gilead Sciences Inc.; (Foster City, CA, USA) Arcus Biosciences (Hayward, CA, USA)	Combination therapy–anti-TROP2 ADC (Topoisomerase I–based ADC) + adenosine-pathway inhibitor (CD39/CD73/A2A/A2B inhibitors)	Combination concept pairing topoisomerase I–based ADCs with inhibitors of the adenosine signaling axis (CD39/CD73/A2A/A2B) to explore integrated cytotoxic and immunometabolic modulation in TROP2-expressing TNBC	Early translational/preclinical platform	Moderate–High (immune–cytotoxic synergy)	Published/Active (patent application)	[126]
TWI816881B	2023	Hengyi Biopharma (Shanghai) Co., Ltd. (Shanghai, China)	Combination therapy–BET bromodomain inhibitor (Compound I) + PARP inhibitor (±checkpoint inhibitor)	Combination of a BET bromodomain inhibitor with a PARP inhibitor, proposed to induce HRD-like states in BRCA-wild-type TNBC, with optional expansion to immune checkpoint blockade	Early clinical/translational	Moderate (PARPi expansion)	Active	[127]
US11001628B2	2021	Novartis AG (Basel, Switzerland)	Antibody combination: anti-PD-1 + anti-M-CSF (dual-pathway immunotherapy)	Conceptual combination of PD-1 blockade with inhibition of the M-CSF signaling pathway, designed to address macrophage-mediated immunosuppression alongside T-cell reactivation	Translational/early clinical	Moderate (resistance reversal)	Expired–Fee Related (expired due to non-payment of maintenance fees; the technology is no longer under patent protection)	[128]

Abbreviations: TNBC—triple-negative breast cancer; ADC—antibody–drug conjugate; DXd—exatecan-derived topoisomerase I inhibitor payload; PARP—poly(ADP-ribose) polymerase; DDR—DNA damage response; TROP2—trophoblast cell-surface antigen 2; CD39/CD73/A2A/A2B—components of the adenosine immunosuppressive pathway; BET—bromodomain and extra-terminal motif protein; PD-1—programmed death-1; M-CSF—macrophage colony-stimulating factor; BRCA—breast cancer susceptibility genes.

## Data Availability

The original contributions presented in this study are included in the article. Further inquiries can be directed to the corresponding authors.
